# Id2 epigenetically controls CD8^+^ T-cell exhaustion by disrupting the assembly of the Tcf3-LSD1 complex

**DOI:** 10.1038/s41423-023-01118-6

**Published:** 2024-01-29

**Authors:** Yiming Li, Mingwei Han, Haolin Wei, Wan Huang, Zhinan Chen, Tianjiao Zhang, Meirui Qian, Lin Jing, Gang Nan, Xiuxuan Sun, Shuhui Dai, Kun Wang, Jianli Jiang, Ping Zhu, Liang Chen

**Affiliations:** 1 Department of Cell Biology of National Translational Science Center for Molecular Medicine and Department of Clinical Immunology of Xijing Hospital, Fourth Military Medical University, Xi’an, Shaanxi 710032 China; 2 State Key Laboratory of New Targets Discovery and Drug Development for Major Diseases, Ganzhou, Jiangxi, 341000, Xi’an, Shaanxi 710032 China; 3 School of Medicine, Shanghai University, Shanghai, 200444 China

**Keywords:** Id2, T-cell exhaustion, Epigenetic modification, Immune evasion, Oncology, Immunology

## Abstract

CD8^+^ T-cell exhaustion is a state of dysfunction that promotes tumor progression and is marked by the generation of Slamf6^+^ progenitor exhausted (Tex^prog^) and Tim-3^+^ terminally exhausted (Tex^term^) subpopulations. Inhibitor of DNA binding protein 2 (Id2) has been shown to play important roles in T-cell development and CD8^+^ T-cell immunity. However, the role of Id2 in CD8^+^ T-cell exhaustion is unclear. Here, we found that Id2 transcriptionally and epigenetically regulates the generation of Tex^prog^ cells and their conversion to Tex^term^ cells. Genetic deletion of Id2 dampens CD8^+^ T-cell-mediated immune responses and the maintenance of stem-like CD8^+^ T-cell subpopulations, suppresses PD-1 blockade and increases tumor susceptibility. Mechanistically, through its HLH domain, Id2 binds and disrupts the assembly of the Tcf3-Tal1 transcriptional regulatory complex, and thus modulates chromatin accessibility at the Slamf6 promoter by preventing the interaction of Tcf3 with the histone lysine demethylase LSD1. Therefore, Id2 increases the abundance of the permissive H3K4me2 mark on the Tcf3-occupied E-boxes in the Slamf6 promoter, modulates chromatin accessibility at the Slamf6 promoter and epigenetically regulates the generation of Slamf6^+^ Tex^prog^ cells. An LSD1 inhibitor GSK2879552 can rescue the Id2 knockout phenotype in tumor-bearing mice. Inhibition of LSD1 increases the abundance of Slamf6^+^Tim-3^−^ Tex^prog^ cells in tumors and the expression level of Tcf1 in Id2-deleted CD8^+^ T cells. This study demonstrates that Id2-mediated transcriptional and epigenetic modification drives hierarchical CD8^+^ T-cell exhaustion, and the mechanistic insights gained may have implications for therapeutic intervention with tumor immune evasion.

## Introduction

T-cell exhaustion is a state of dysfunction acquired during chronic viral infections and cancer [1, 2]. During chronic viral infections, exhausted CD8^+^ T cells hierarchically lose their functional properties, such as cytokine production and proliferative and cytolytic capacities, accompanied by upregulation of multiple inhibitory receptors and retention of memory potential, which mirror those observed in cancer [3–6].

Two subpopulations of exhausted CD8^+^ T cells have been identified in chronic viral infections and cancer, each with distinct functional properties and epigenetic landscapes. Progenitor or stem-like exhausted CD8^+^ T cells (Tex^prog^ cells) are defined by a programmed cell death (PD)-1^int^, Slamf6^+^ or CXCR5^+^ signature and are characterized by polyfunctional cytokine production, boosted proliferative capacity, long-term persistence and the potential for conversion to terminally exhausted cells [7–10]. Terminally exhausted T cells (Tex^term^ cells), identified by a PD-1^hi^ or Tim-3^+^ signature, exhibit an enhanced cytolytic capacity despite their reduced polyfunctional cytokine production, proliferative capacity and longevity [7, 9, 10]. Anti-PD-1 therapy increases the number of Tex^prog^ cells and promotes their conversion to Tex^term^ cells [8, 10]. Consequently, the Tex^prog^ subpopulation can respond to immune checkpoint blockade (ICB) therapy and restrain tumor growth more efficiently than the Tex^term^ subpopulation. Moreover, the Tex^prog^ subpopulation accounted for a larger proportion of T cells in melanoma patients who experienced a durable response to ICB [8, 11]. Thus, approaches to increase the proportion of the Tex^prog^ subpopulation may promote the efficacy of ICB. However, the mechanisms underlying the generation and conversion of CD8^+^ Tex cells remain unknown.

Dysregulation of transcriptional and epigenetic programs drives aberrant antitumor immune responses of CD8^+^ Tex cells [12]. Thus far, the transcriptional and epigenetic pathways that drive Tex^prog^-to-Tex^term^ conversion remain undefined. In addition, the specialized chromatin-modifying enzymes that act as epigenetic ‘writers’ or ‘erasers’ to mediate CD8^+^ T-cell dysfunction are unknown. Therefore, it is crucial to investigate the complex interplay between epigenetics and immunology in the context of cancer.

In the present study, we show that inhibitor of DNA binding protein 2 (Id2) transcriptionally and epigenetically promotes the generation of Slamf6^+^ Tex^prog^ cells and their conversion to Tex^term^ cells. Therefore, Id2 restrains tumor immune evasion. Genetic deletion of Id2 in T cells promotes tumor development by suppressing the immune response and represses the tumor response to PD-1 blockade. Id2 ablation inhibits the expansion and cytokine production of therapeutic CD8^+^ T cells and attenuates the maintenance of stem cell-like CD8^+^ T cells after T-cell therapy. Furthermore, Id2 regulates the generation of Slamf6^+^ Tex^prog^ cells and their conversion to Tex^term^ cells in tumors. Mechanistically, Id2 binds and disrupts the Tcf3-Tal1 transcriptional regulatory complex through its helix-loop-helix (HLH) domain and alters the chromatin accessibility of *Slamf6*. Deletion of the HLH domain in Id2 allows Tcf3 to recruit and bind lysine-specific demethylase 1 (LSD1), a histone lysine demethylase. As a result, the Tcf3-LSD1 complex removes the permissive histone H3 lysine 4 dimethylation (H3K4me2) mark from Tcf3-occupied E-boxes in the *Slamf6* promoter and thus regulates the Tex^prog^-to-Tex^term^ conversion. Our study demonstrates that Id2 drives T-cell exhaustion in a transcriptional and epigenetic manner and might be harnessed for therapeutic interventions to reverse T-cell exhaustion and restrain tumor progression.

## Results

### Id2 is selectively upregulated in tumor-infiltrating CD8^+^ T cells

**Fig. 1:**
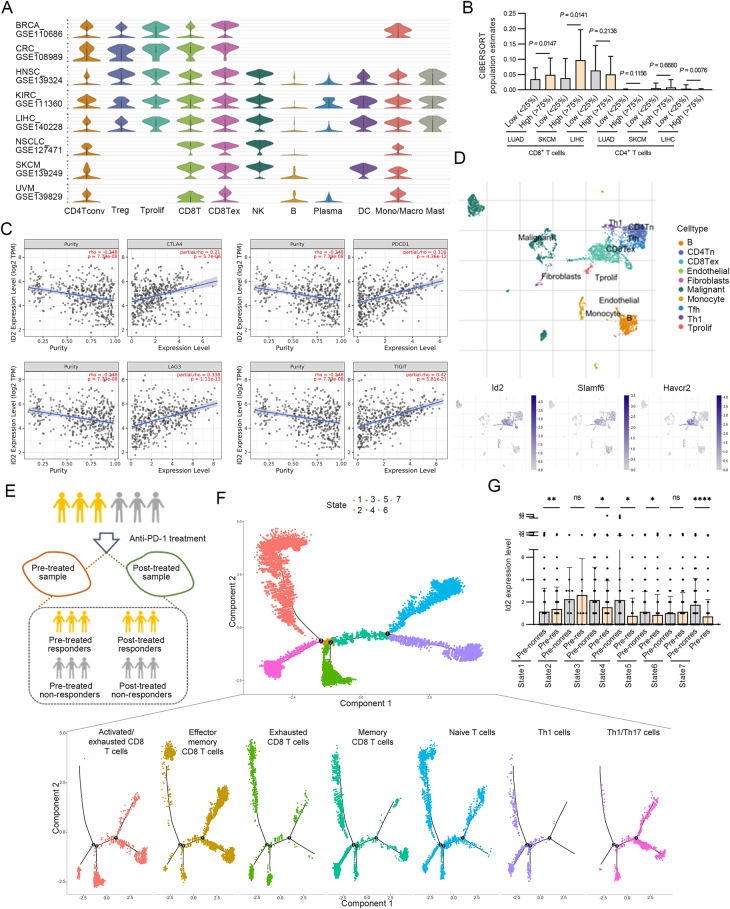
Id2 is selectively upregulated in tumor-infiltrating CD8^+^ T cells. **A** Distribution of Id2 expression in different immune cell types across datasets (BRCA_GSE110686, CRC_GSE108989, HNSC_GSE139324, KIRC_GSE111360, LIHC_GSE140228_10X, NSCLC_GSE127471, SKCM_GSE139249, and UVM_GSE139829). CD4Tconv, conventional CD4^+^ T cells; Treg, regulatory T cells; Tprolif, proliferative T cells; CD8T, CD8^+^ T cells; CD8Tex, exhausted CD8^+^ T cells; NK, natural killer cells; B, B cells; plasma, plasma cells; DC dendritic cells, Mono/Macro monocytes or macrophages, Mast mast cells. **B** Relative abundances of tumor-infiltrating immune cell populations determined by the CIBERSORT tool in LUAD, SKCM and LIHC patients with varying Id2 expression levels based on RNA-seq data in TCGA. LUAD, lung adenocarcinoma, *n* = 532; SKCM, skin cutaneous melanoma, *n* = 102; LIHC, liver hepatocellular carcinoma, *n* = 370. **C** Correlations of *Id2* expression with the expression of inhibitory receptors (*Pdcd1*, *Ctla4*, *Lag3* and *Tigit*) in SKCM. The degree of correlation is the purity-adjusted partial Spearman’s rho value. **D** Id2 expression profiles visualized on a tSNE plot of major immune cell types in the SKCM TME. The GSE72056 dataset contains data from 19 patients (4645 cells). **E** Schematic of the procedure for single-cell RNA-seq analysis of samples obtained before and after anti-PD-1 therapy in patients with basal cell carcinoma (GSE123813). **F** Trajectory analysis of T-cell subsets in responders and nonresponders. **G** Id2 expression levels in seven states in responders and nonresponders. ns, not significant; ****P < 0.0001; **P < 0.01; *P < 0.05

To identify associations between Id2 expression and inferred immune infiltration in human tumors, we investigated the Tumor Immune Single-cell Hub (TISCH) database, a single-cell RNA-seq (scRNA-seq) database focusing on the tumor microenvironment (TME). We found that Id2 was selectively upregulated in proliferative T (Tprolif) cells, CD8^+^ T (CD8T) cells, exhausted CD8^+^ T (CD8Tex) cells and natural killer (NK) cells, especially in CD8T and CD8Tex cells (Fig. 1A). Furthermore, we estimated the relative abundances of immune cell populations using the CIBERSORT tool [13] and bulk tumor RNA-seq data of lung adenocarcinoma (LUAD), skin cutaneous melanoma (SKCM) and liver hepatocellular carcinoma (LIHC) from The Cancer Genome Atlas. Consistent with the findings in the TISCH database, a high expression level of Id2 was associated with an increased population of CD8^+^ T cells, especially in LUAD and SKCM (Fig. 1B and Supplementary Fig. 1A). The TME can upregulate the expression of many inhibitory receptors and subvert immune surveillance to favor immune evasion [14, 15]. We found that the expression of Id2 was highly correlated with that of many inhibitory receptors (*Pdcd1*, *Ctla4*, *Havcr2*, *Lag3, Klrc1*, *Cd244*, etc.) in SKCM (Fig. 1C and Supplementary Fig. 1B). TISCH scRNA-seq data demonstrated that Id2 exhibited an obvious overlapping distribution in CD8^+^ Tex cells, along with Havcr2 and Slamf6, two markers indicating terminal and progenitor cells, respectively, in both the overall SKCM and anti-PD-1-treated SKCM subgroups (Fig. 1D and Supplementary Fig. 1C). Furthermore, we analyzed the scRNA-seq data of samples obtained from patients with basal cell carcinoma before and after anti-PD-1 therapy (Fig. 1E). We found that the evolutionary trajectories of the major T-cell subsets proceeded through seven states, with different subsets exhibiting distinct distributions (Fig. 1F). Id2 exhibited significantly higher expression levels in activated/exhausted CD8^+^ T cells in pretreated samples from responders to anti-PD-1 treatment than in the counterpart samples from nonresponders (Fig. 1G). In addition, we found that patients with SKCM achieved the best cumulative survival outcomes when they had both a high expression level of Id2 and high infiltration of CD8^+^ T cells (Supplementary Fig. 1D). These results indicate that Id2 may play a critical role in the T-cell-mediated immune response in tumors.

### Deletion of Id2 in T cells promotes tumor development by suppressing the immune response

**Fig. 2:**
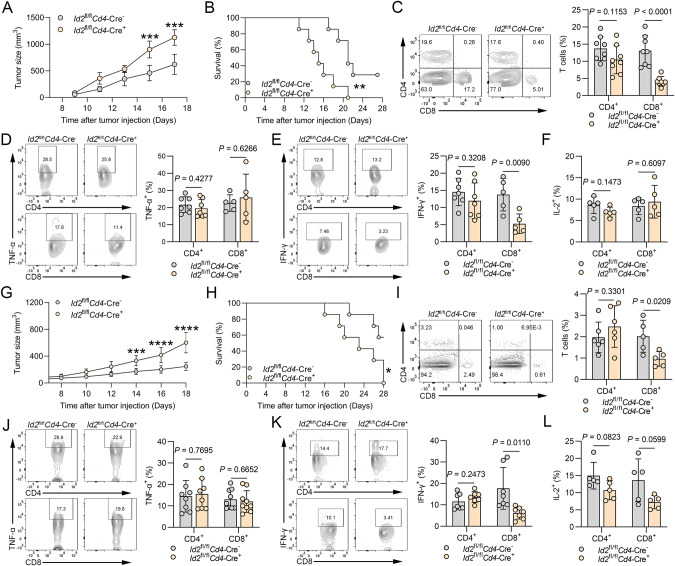
Id2 ablation in T cells promotes tumor development by suppressing the immune response. **A** Tumor growth in *Id2*^fl/fl^*Cd4*-Cre^-^ mice and *Id2*^fl/fl^*Cd4*-Cre^+^ mice injected subcutaneously (s.c.) with 2 × 10^5^ B16-F10 melanoma cells. ***P < 0.001. **B** Survival curves of *Id2*^fl/fl^*Cd4*-Cre^-^ mice and *Id2*^fl/fl^*Cd4*-Cre^+^ mice injected subcutaneously (s.c.) with 2 × 10^5^ B16-F10 melanoma cells. **P < 0.01. **C** Flow cytometric analysis of the frequencies of CD4^+^ T cells and CD8^+^ T cells in the tumors of *Id2*^fl/fl^*Cd4*-Cre^-^ mice and *Id2*^fl/fl^*Cd4*^-^Cre^+^ mice injected s.c. with B16-F10 melanoma cells (Day 14). The data are presented as representative plots and summary graphs. **D** Flow cytometric analysis of the frequencies of TNF-α-expressing CD4^+^ T cells and CD8^+^ T cells in the tumors of *Id2*^fl/fl^*Cd4*-Cre^-^ mice and *Id2*^fl/fl^*Cd4*^-^Cre^+^ mice injected s.c. with B16-F10 melanoma cells (Day 14). The data are presented as representative plots and summary graphs. **E** Flow cytometric analysis of the frequencies of IFN-γ-expressing CD4^+^ T cells and CD8^+^ T cells in the tumors of *Id2*^fl/fl^*Cd4*-Cre^-^ mice and *Id2*^fl/fl^*Cd4*^-^Cre^+^ mice injected s.c. with B16-F10 melanoma cells (Day 14). The data are presented as representative plots and summary graphs. **F** Summary graphs of IL-2-expressing CD4^+^ T cells and CD8^+^ T cells in the tumors of *Id2*^fl/fl^*Cd4*-Cre^-^ mice and *Id2*^fl/fl^*Cd4*^-^Cre^+^ mice injected s.c. with B16-F10 melanoma cells (Day 14). **G** Tumor growth in *Id2*^fl/fl^*Cd4*-Cre^-^ mice and *Id2*^fl/fl^*Cd4*^-^Cre^+^ mice injected subcutaneously (s.c.) with 2 × 10^6^ LLC lung cancer cells. ***P < 0.001; ****P < 0.0001. **H** Survival curves of *Id2*^fl/fl^*Cd4*-Cre^-^ mice and *Id2*^fl/fl^*Cd4*-Cre^+^ mice injected subcutaneously (s.c.) with 2 × 10^6^ LLC lung cancer cells. *P < 0.05. **I** Flow cytometric analysis of the frequencies of CD4^+^ T cells and CD8^+^ T cells in the tumors of *Id2*^fl/fl^*Cd4*^-^Cre^-^ mice and *Id2*^fl/fl^*Cd4*-Cre^+^ mice injected s.c. with LLC lung cancer cells (Day 14). The data are presented as representative plots and summary graphs. **J** Flow cytometric analysis of the frequencies of TNF-α-expressing CD4^+^ T cells and CD8^+^ T cells in the tumors of *Id2*^fl/fl^*Cd4*^-^Cre^-^ mice and *Id2*^fl/fl^*Cd4*-Cre^+^ mice injected s.c. with LLC lung cancer cells (Day 14). The data are presented as representative plots and summary graphs. **K** Flow cytometric analysis of the frequencies of IFN-γ-expressing CD4^+^ T cells and CD8^+^ T cells in the tumors of *Id2*^fl/fl^*Cd4*^-^Cre^-^ mice and *Id2*^fl/fl^*Cd4*-Cre^+^ mice injected s.c. with LLC lung cancer cells (Day 14). The data are presented as representative plots and summary graphs. **L** Summary graphs of IL-2-expressing CD4^+^ T cells and CD8^+^ T cells in the tumors of *Id2*^fl/fl^*Cd4*^-^Cre^-^ mice and *Id2*^fl/fl^*Cd4*-Cre^+^ mice injected s.c. with LLC lung cancer cells (Day 14)

To assess the role of Id2 in T cells, we crossed *Id2*^fl/fl^ mice with *Cd4*-Cre mice to obtain mice with conditional *Id2* knockout in T cells (*Id2*^fl/fl^*Cd4*-Cre mice) (Supplementary Fig. 2A). *Id2*^fl/fl^*Cd4*-Cre^+^ mice expressed the Cre transgene under the control of the *Cd4*-Cre sequence, which resulted in Id2 deletion in CD4^+^CD8^+^ thymocytes. Since mature CD4^+^ and CD8^+^ T cells are produced from CD4^+^CD8^+^ progenitor cells via positive selection in the thymus, Id2 was deleted in both CD4^+^ and CD8^+^ T cells in *Id2*^fl/fl^*Cd4*-Cre^+^ mice. We first examined the composition of naïve CD4^+^ and CD8^+^ T cells in the spleens and lymph nodes of *Id2*^fl/fl^*Cd4*-Cre^+^ mice and their littermates and found that *Id2*^fl/fl^*Cd4*-Cre^+^ mice and their littermates had similar percentages of these cells both without (Supplementary Fig. 2B) and with PMA stimulation (Supplementary Fig. 2C). Compared with their littermates, *Id2*^fl/f*l*^*Cd4*-Cre^+^ mice showed lower percentages of several CD8^+^ subsets; for instance, the CD44^+^CD62L^−^ subset, which corresponds to the effector memory phenotype, was less abundant in *Id2*^fl/fl^*Cd4*-Cre^+^ mice than in their littermates (Supplementary Fig. 2D). To determine whether loss of Id2 in T cells affects host antitumor immunity, we analyzed the role of Id2 in antitumor responses using melanoma, Lewis lung cancer and hepatoma models. Compared to the *Id2*^fl/fl^*Cd4*-Cre^−^ mice, the *Id2*^fl/fl^*Cd4*-Cre^+^ mice exhibited a dramatic increase in tumor growth, with premature lethality (Fig. 2A, B; Supplementary Fig. 2E). The melanoma-bearing *Id2*^fl/fl^*Cd4*-Cre^+^ mice had a dramatically decreased frequency of tumor-infiltrating CD8^+^ T cells, but the frequency of CD4^+^ T cells was normal (Fig. 2C). Given that exhausted T cells showed impaired cytokine production, we investigated the expression levels of TNF-α, IFN-γ and IL-2 in tumor-infiltrating T cells. Decreased IFN-γ expression was observed in tumor-infiltrating CD8^+^ T cells from the *Id2*^fl/fl^*Cd4*-Cre^+^ mice in comparison with their *Id2*^fl/fl^*Cd4*-Cre^−^ littermates, while no such difference was observed in CD4^+^ T cells (Fig. 2E). In addition, no significant difference was observed in the frequency of tumor-infiltrating TNF-α-expressing (Fig. 2D) or IL-2-expressing (Fig. 2F) T cells between the *Id2*^fl/fl^*Cd4*-Cre^−^ mice and *Id2*^fl/fl^*Cd4*-Cre^+^ mice. Parallel studies were also conducted in a Lewis lung cancer model. Consistent with the above findings, increased tumor growth and premature lethality were observed in the *Id2*^fl/fl^*Cd4*-Cre^+^ mice compared to their *Id2*^fl/fl^*Cd4*-Cre^−^ littermates (Fig. 2G, H). A significantly decreased frequency of tumor-infiltrating CD8^+^ T cells was observed in the *Id2*^fl/fl^*Cd4*-Cre^+^ mice, but the frequency of CD4^+^ T cells was normal (Fig. 2I). Decreased IFN-γ expression was observed in tumor-infiltrating CD8^+^ T cells from the *Id2*^fl/fl^*Cd4*-Cre^+^ mice compared to the *Id2*^fl/fl^*Cd4*-Cre^−^ mice, while no such difference was observed in CD4^+^ T cells (Fig. 2K). In addition, no significant difference was observed in the frequency of tumor-infiltrating TNF-α-expressing (Fig. 2J) or IL-2-expressing (Fig. 2L) T cells between the *Id2*^fl/fl^*Cd4*-Cre^−^ mice and *Id2*^fl/fl^*Cd4*-Cre^+^ mice. These results indicate that Id2 in CD8^+^ T cells may play a crucial role in antitumor immune responses in vivo. Next, we evaluated T-cell-mediated cytotoxicity in a coculture system of murine Hepa1-6^GFP^-SIINFEKL cells and OT-I CD8^+^ T cells. These Hepa1-6 hepatoma cells expressed the chicken ovalbumin OVA_257-264_ (SIINFEKL) peptide, which can endow antigen-specific OT-I CD8^+^ T cells with cytolytic capacity upon recognition of SIINFEKL peptides presented by H2-K^b^ molecules. We found that *Id2*^fl/fl^*Cd4*-Cre^+^ OT-I CD8^+^ T cells endowed Hepa1-6^GFP^-SIINFEKL cells with resistance to T-cell-mediated cytotoxicity (Supplementary Fig. 2F). Taken together, these results suggest that Id2 in T cells prevents tumor development by enhancing the antigen-specific CD8^+^ T-cell immune response.

**Fig. 3:**
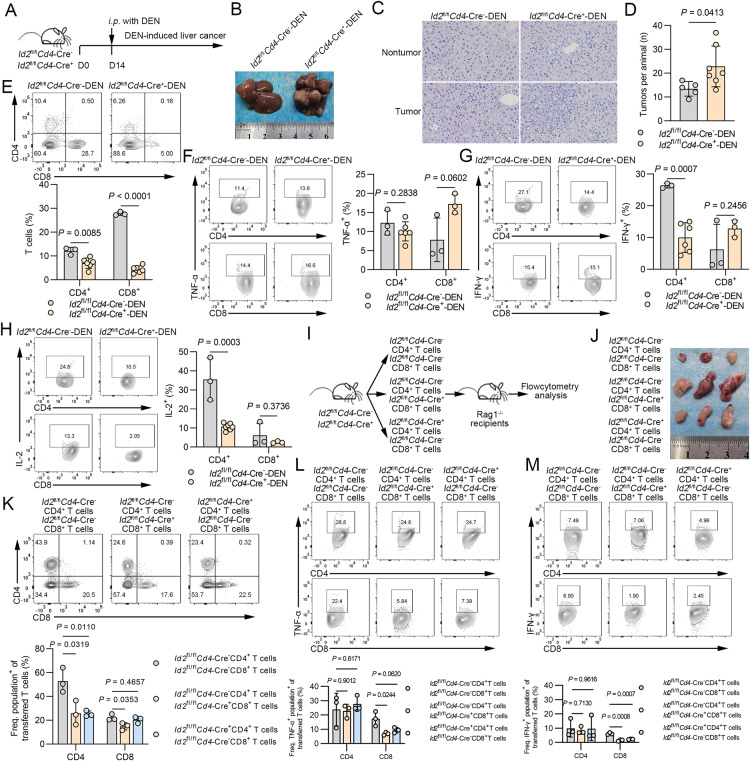
Id2 ablation restrains T-cell-mediated immune surveillance in both DEN-induced and murine models of hepatoma. **A** *Id2*^fl/fl^*Cd4*-Cre^−^ mice and *Id2*^fl/fl^*Cd4*-Cre^+^ mice were administered diethylnitrosamine (DEN) on postnatal day 14 and sacrificed after 40 weeks. **B**, **C** Macroscopic and microscopic evaluations of tumor formation were conducted 40 weeks after DEN injection. **D** The tumor numbers were assessed in every liver lobe. **E** Flow cytometric analysis of the frequencies of hepatic CD4^+^ T cells and CD8^+^ T cells in DEN-induced tumors of *Id2*^fl/fl^*Cd4*-Cre^−^ mice and *Id2*^fl/fl^*Cd4*-Cre^+^ mice. The data are presented as representative plots and summary graphs. **F** Flow cytometric analysis of the frequencies of hepatic TNF-α-expressing CD4^+^ T cells and CD8^+^ T cells in DEN-induced tumors of *Id2*^fl/fl^*Cd4*-Cre^−^ mice and *Id2*^fl/fl^*Cd4*-Cre^+^ mice. All cells derived from tumors were stimulated with phorbol 12-myristate 13-acetate (PMA) and ionomycin for 6 h and then subjected to intracellular staining. The data are presented as representative plots and summary graphs. **G** Flow cytometric analysis of the frequencies of hepatic IFN-γ-expressing CD4^+^ T cells and CD8^+^ T cells in DEN-induced tumors of *Id2*^fl/fl^*Cd4*-Cre^−^ mice and *Id2*^fl/fl^*Cd4*-Cre^+^ mice. All cells derived from tumors were stimulated with phorbol 12-myristate 13-acetate (PMA) and ionomycin for 6 h and then subjected to intracellular staining. The data are presented as representative plots and summary graphs. **H** Flow cytometric analysis of the frequencies of hepatic IL-2-expressing CD4^+^ T cells and CD8^+^ T cells in DEN-induced tumors of *Id2*^fl/fl^*Cd4*-Cre^−^ mice and *Id2*^fl/fl^*Cd4*-Cre^+^ mice. All cells derived from tumors were stimulated with phorbol 12-myristate 13-acetate (PMA) and ionomycin for 6 h and then subjected to intracellular staining. The data are presented as representative plots and summary graphs. **I** Schematic of the analysis of Rag1^−/−^ mice injected s.c. with 2 × 10^6^ Hepa1-6 hepatoma cells after adoptive transfer of 2 × 10^6^ *Id2*^fl/fl^*Cd4*-Cre^−^ or *Id2*^fl/fl^*Cd4*-Cre^+^ CD8^+^ T cells along with 2 × 10^6^ *Id2*^fl/fl^*Cd4*-Cre^−^ CD4^+^ T cells. **J** Gross assessment of tumors in the Rag1^−/−^ model. **K** Flow cytometric analysis of the frequencies of adoptive CD4^+^ T cells and CD8^+^ T cells in the tumors of Rag1^−/−^ mice injected s.c. with Hepa1-6 hepatoma cells (Day 14). The data are presented as representative plots and summary graphs. **L** Flow cytometric analysis of the frequencies of adoptive TNF-α-expressing CD4^+^ T cells and CD8^+^ T cells in the tumors of Rag1^−/−^ mice injected s.c. with Hepa1-6 hepatoma cells (Day 14). All cells derived from Hepa1-6 tumors were stimulated with phorbol 12-myristate 13-acetate (PMA) and ionomycin for 6 h and then subjected to intracellular staining. The data are presented as representative plots and summary graphs. **M** Flow cytometric analysis of the frequencies of adoptive IFN-γ-expressing CD4^+^ T cells and CD8^+^ T cells in the tumors of Rag1^−/−^ mice injected s.c. with Hepa1-6 hepatoma cells (Day 14). All cells derived from Hepa1-6 tumors were stimulated with phorbol 12-myristate 13-acetate (PMA) and ionomycin for 6 h and then subjected to intracellular staining. The data are presented as representative plots and summary graphs

In addition to performing studies in the above murine tumor models, we investigated tumor-induced immune responses using a murine diethylnitrosamine (DEN)-induced liver cancer model. This model features genetic heterogeneity and strong immunogenicity, thus resembling the lethal hepatocellular carcinoma phenotype in humans [16]. To assess the role of Id2 in hepatoma development and progression, we administered DEN to *Id2*^fl/fl^*Cd4*-Cre^−^ mice and *Id2*^fl/fl^*Cd4*-Cre^+^ mice on postnatal day 14 and monitored tumor development in the following 40 weeks (Fig. 3A). An elevated tumor burden and severe cellular atypia were found in the *Id2*^fl/fl^*Cd4*-Cre^+^ mice (Fig. 3B, C). In addition, an increased tumor number was observed in the *Id2*^fl/fl^*Cd4*-Cre^+^ mice compared to their *Id2*^fl/fl^*Cd4*-Cre^−^ littermates (Fig. 3D). Furthermore, we assessed the immune microenvironment of DEN-induced tumors. Decreased frequencies of both tumor-infiltrating CD4^+^ and CD8^+^ T cells were observed in the *Id2*^fl/fl^*Cd4*-Cre^+^ mice compared to the *Id2*^fl/fl^*Cd4*-Cre^−^ mice (Fig. 3E). Decreased IFN-γ and IL-2 levels were observed in tumor-infiltrating CD4^+^ T cells from the *Id2*^fl/fl^*Cd4*-Cre^+^ mice compared with the *Id2*^fl/fl^*Cd4*-Cre^−^ mice, while no such differences were observed in CD8^+^ T cells (Fig. 3G, H). No significant differences were observed in the frequencies of TNF-α-expressing tumor-infiltrating CD4^+^ and CD8^+^ T cells (Fig. 3F). These results indicate that Id2 ablation restrains T-cell-mediated immune surveillance of malignant hepatocytes in DEN-induced tumors. Next, we reconstituted Rag1^−/−^ mice with *Id2*^fl/fl^*Cd4*-Cre^−^ or *Id2*^fl/fl^*Cd4*-Cre^+^ CD8^+^ T cells along with the corresponding *Id2*^fl/fl^*Cd4*-Cre^−^ CD4^+^ T cells (Fig. 3I). After Hepa1-6 cell injection, Rag1^−/−^ mice reconstituted with *Id2*^fl/fl^*Cd4*-Cre^+^ CD8^+^ T cells along with *Id2*^fl/fl^*Cd4*-Cre^−^ CD4^+^ T cells displayed increased tumor susceptibility, as shown by the elevated tumor burden (Fig. 3J). We further examined cytokine production in the reconstituted inflammatory tumor microenvironment. Rag1^−/−^ mice challenged with *Id2*^fl/fl^*Cd4*-Cre^+^ CD8^+^ T cells plus *Id2*^fl/fl^*Cd4*-Cre^−^ CD4^+^ T cells had significantly decreased frequencies of adoptive tumor-infiltrating CD8^+^ T cells and CD4^+^ T cells (Fig. 3K). Decreased TNF-α (Fig. 3L) and IFN-γ (Fig. 3M) expression was observed in adoptively transferred tumor-infiltrating CD8^+^ T cells in Rag1^−/−^ mice challenged with *Id2*^fl/fl^*Cd4*-Cre^+^ CD8^+^ T cells plus *Id2*^fl/fl^*Cd4*-Cre^−^ CD4^+^ T cells. In addition, mice adoptively transferred with a single T-cell population, especially those receiving CD8^+^ T cells, showed potent antitumor immunity (Supplementary Fig. 2G). We also examined the abundance of Tregs in tumors and observed no significant difference between *Id2*^fl/fl^*Cd4*-Cre^+^ mice and their littermates (Supplementary Fig. 2H). In addition, we assessed the expression levels of major chemokine receptors, such as CCR7 and CX3CR1, in CD8^+^ T cells but found no obvious differences between *Id2*^fl/fl^*Cd4*-Cre^+^ and *Id2*^fl/fl^*Cd4*-Cre^−^ OT-I CD8^+^ T cells, suggesting that T-cell migration and infiltration may not be disturbed in vivo in the absence of Id2 (Supplementary Fig. 2I). Collectively, these findings suggest that Id2 enhances the host T-cell antitumor immune response.

### Id2 ablation inhibits the expansion and cytokine production of therapeutic CD8^+^ T cells

**Fig. 4:**
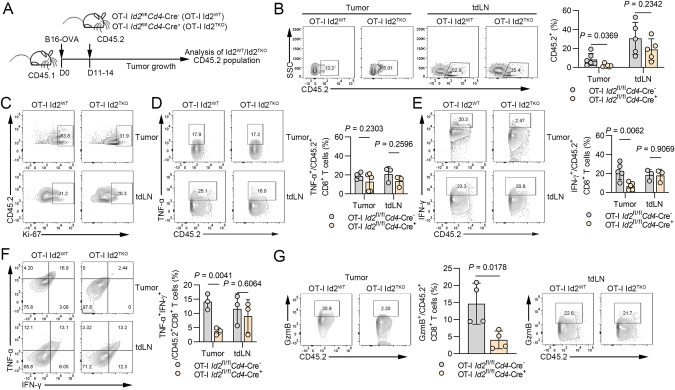
Id2 ablation inhibits the expansion and cytokine production of therapeutic CD8^+^ T cells. **A** Schematic of the analysis of tumor-bearing mice after OT-I CD8^+^ T-cell transfer. Tumor and tumor-draining lymph nodes (tdLNs) were analyzed 11 to 14 days after T-cell therapy. **B** Flow cytometric analysis of the frequencies of adoptive CD8^+^ T cells in *Id2*^fl/fl^*Cd4*-Cre^-^ mice and *Id2*^fl/fl^*Cd4*-Cre^+^ mice in B16-OVA tumors and tdLNs. The data are presented as representative plots and summary graphs. **C** Representative flow cytometric plots of the frequencies of adoptive Ki-67-expressing CD8^+^ T cells of *Id2*^fl/fl^*Cd4*-Cre^-^ mice and *Id2*^fl/fl^*Cd4*-Cre^+^ mice in B16-OVA tumors and tdLNs. **D** Flow cytometric analysis of the frequencies of adoptive TNF-α-expressing CD8^+^ T cells in *Id2*^fl/fl^*Cd4*-Cre^-^ mice and *Id2*^fl/fl^*Cd4*-Cre^+^ mice in B16-OVA tumors and tdLNs. The data are presented as representative plots and summary graphs. **E** Flow cytometric analysis of the frequencies of adoptive IFN-γ-expressing CD8^+^ T cells in *Id2*^fl/fl^*Cd4*-Cre^-^ mice and *Id2*^fl/fl^*Cd4*-Cre^+^ mice in B16-OVA tumors and tdLNs. The data are presented as representative plots and summary graphs. **F** Flow cytometric analysis of the frequencies of adoptive TNF-α^+^IFN-γ^+^ CD8^+^ T cells in *Id2*^fl/fl^*Cd4*-Cre^-^ mice and *Id2*^fl/fl^*Cd4*-Cre^+^ mice in B16-OVA tumors and tdLNs. The data are presented as representative plots and summary graphs. **G** Flow cytometric analysis of the frequencies of adoptive granzyme B-expressing CD8^+^ T cells in *Id2*^fl/fl^*Cd4*-Cre^-^ mice and *Id2*^fl/fl^*Cd4*-Cre^+^ mice in B16-OVA tumors and tdLNs. The data are presented as representative plots and summary graphs

To confirm the role of Id2 in antigen-specific CD8^+^ T-cell activity, we adoptively transferred CD8^+^ T cells sorted from *Id2*^fl/fl^*Cd4*-Cre^+^ OT-I mice and their *Id2*^fl/fl^*Cd4*-Cre^−^ OT-I littermates into B16-OVA tumor-bearing B6.SJL (CD45.1) mice and then tracked and detected donor and host T cells based on the congenic markers CD45.2 and CD45.1, respectively (Fig. 4A). Deletion of Id2 resulted in a decreased frequency of adoptive OVA-specific CD8^+^ T cells in tumors (Fig. 4B). Id2 ablation also led to decreased frequencies of adoptive Ki-67-expressing OVA-specific CD8^+^ T cells in tumors and tumor-draining lymph nodes (tdLNs) (Fig. 4C). To investigate the role of Id2 in cytokine production, we analyzed the production of TNF-α, IFN-γ and granzyme B in adoptively transferred OT-I CD8^+^ T cells in tumors and tdLNs. Consistent with the above results, Id2 ablation decreased the frequencies of adoptive IFN-γ-expressing OVA-specific CD8^+^ T cells and TNF-α^+^IFN-γ^+^ OVA-specific CD8^+^ T cells in tumors, whereas no difference was observed in tdLNs (Fig. 4E, F). Similarly, Id2 deficiency did not alter the production of TNF-α in adoptive OVA-specific CD8^+^ T cells (Fig. 4D). Moreover, loss of Id2 resulted in a decreased frequency of adoptive granzyme B-expressing OVA-specific CD8^+^ T cells in tumors (Fig. 4G). Together, these results suggest that Id2 is required for the proliferation and effector function of tumor-infiltrating CD8^+^ T cells, thereby facilitating tumor eradication.

### Id2 sustains the maintenance of stem cell-like CD8^+^ T cells after T cell transfer therapy

**Fig. 5:**
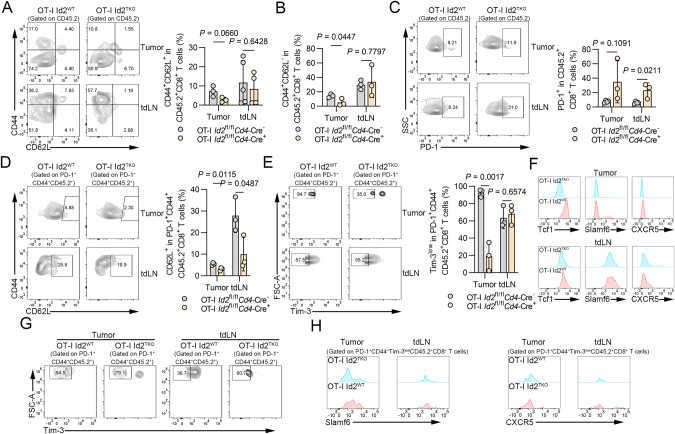
Id2 ablation attenuates the maintenance of stem cell-like CD8^+^ T cells after T-cell therapy. **A**, **B** Flow cytometric analysis of the frequencies of adoptive CD44^+^CD62L^+^ and CD44^+^CD62L^-^ CD8^+^ T cells in B16-OVA tumors and tdLNs of *Id2*^fl/fl^*Cd4*-Cre^-^ mice and *Id2*^fl/fl^*Cd4*-Cre^+^ mice. The data are presented as representative plots and summary graphs. **C** Flow cytometric analysis of the frequencies of adoptive PD-1-expressing CD8^+^ T cells in B16-OVA tumors and tdLNs of *Id2*^fl/fl^*Cd4*-Cre^-^ mice and *Id2*^fl/fl^*Cd4*-Cre^+^ mice. The data are presented as representative plots and summary graphs. **D** Flow cytometric analysis of the frequencies of adoptive CD62L-expressing CD8^+^ T cells gated on PD-1^+^CD44^+^CD45.2^+^ cells in B16-OVA tumors and tdLNs of *Id2*^fl/fl^*Cd4*-Cre^-^ mice and *Id2*^fl/fl^*Cd4*-Cre^+^ mice. The data are presented as representative plots and summary graphs. **E** Flow cytometric analysis of the frequencies of adoptive Tim-3^low^ CD8^+^ T cells gated on PD-1^+^CD44^+^CD45.2^+^ cells in B16-OVA tumors and tdLNs of *Id2*^fl/fl^*Cd4*-Cre^-^ mice and *Id2*^fl/fl^*Cd4*-Cre^+^ mice. The data are presented as representative plots and summary graphs. **F** Representative histograms of Tcf1, Slamf6 and CXCR5 expression in adoptive CD8^+^ T cells in B16-OVA tumors and tdLNs from *Id2*^fl/fl^*Cd4*-Cre^-^ mice and *Id2*^fl/fl^*Cd4*-Cre^+^ mice. **G** Representative flow cytometric plots of adoptive Tim-3^low^ CD8^+^ T cells gated on PD-1^+^CD44^+^CD45.2^+^ cells in B16-OVA tumors and tdLNs of *Id2*^fl/fl^*Cd4*-Cre^-^ mice and *Id2*^fl/fl^*Cd4*-Cre^+^ mice. **H** Representative histograms of Slamf6 or CXCR5 expression in adoptive CD8^+^ T cells gated on PD-1^+^CD44^+^Tim-3^low^CD45.2^+^ cells in B16-OVA tumors and tdLNs from *Id2*^fl/fl^*Cd4*-Cre^-^ mice and *Id2*^fl/fl^*Cd4*-Cre^+^ mice

The exhausted T-cell compartment is heterogeneous. Accumulating evidence reveals that the stem-like exhausted T-cell population serves as a reservoir for the terminally exhausted T-cell population [17, 18]. To assess whether Id2 is necessary for the maintenance of stem-like CD8^+^ T cells, we adoptively transferred IL-15-primed OT-I CD8^+^ T cells into B16-OVA tumor-bearing B6.SJL mice and investigated the properties of OT-I CD8^+^ T cells in tumors and tdLNs. First, we evaluated the memory subpopulations and effector subpopulations of adoptive CD45.2^+^CD8^+^ T cells in tumors and tdLNs based on CD62L and CD44 expression patterns. The CD62L^+^CD44^+^CD8^+^ subset is defined as the central memory subpopulation, which is characterized by robust self-renewal and durable persistence; the CD62L^−^CD44^+^CD8^+^ subset is defined as the effector memory subpopulation, which is characterized by higher production of IFN-γ [19]. Deletion of Id2 resulted in a marginally decreased frequency of tumor-infiltrating central memory cells (CD44^+^CD62L^+^) among adoptive CD45.2^+^ OT-I CD8^+^ T cells from *Id2*^fl/fl^*Cd4*-Cre^+^ OT-I mice compared to those from *Id2*^fl/fl^*Cd4*-Cre^−^ OT-I mice (Fig. 5A). In addition, a decreased frequency of tumor-infiltrating effector memory cells (CD44^+^CD62L^−^) was observed among adoptive CD45.2^+^ OT-I CD8^+^ T cells from *Id2*^fl/fl^*Cd4*-Cre^+^ OT-I mice compared to *Id2*^fl/fl^*Cd4*-Cre^−^ OT-I mice (Fig. 5B). These results indicate that Id2 may account for the maintenance of memory potential in CD8^+^ T cells. Next, we examined the frequencies of PD-1-expressing CD8^+^ T cells among adoptively transferred CD45.2^+^ OT-I CD8^+^ T cells in tumors and tdLNs. Id2 ablation led to a dramatic increase in PD-1 expression in adoptively transferred CD45.2^+^ OT-I CD8^+^ T cells in tdLNs. Likewise, the expression of PD-1 was marginally increased in adoptively transferred CD45.2^+^ OT-I CD8^+^ T cells in tumors (Fig. 5C). These findings indicate that Id2 deficiency may impair T-cell functions in the adoptively transferred cells. Since CD62L is acknowledged as a marker of lymphoid homing and immune memory [20], we assessed CD62L expression in PD-1^+^CD44^+^CD45.2^+^CD8^+^ T cells after adoptive transfer. Consistently, decreased frequencies of CD62L-expressing cells were observed among adoptively transferred PD-1^+^CD44^+^CD45.2^+^ CD8^+^ T cells from *Id2*^fl/fl^*Cd4*-Cre^+^ OT-I mice compared to *Id2*^fl/fl^*Cd4*-Cre^−^ OT-I mice in both tumors and tdLNs (Fig. 5D). Recent studies found that intermediate expression of PD-1 and low expression of Tim-3 were observed on stem-like CD8^+^ T cells, which were identified as PD-1^+^CD44^+^Tim-3^low^ CD8^+^ T cells [18, 21, 22]. Therefore, we assessed the stemness of adoptive PD-1^+^CD44^+^Tim-3^low^ CD8^+^ T cells after T-cell transfer therapy. A dramatically reduced proportion of Tim-3^low^ cells was observed among adoptive PD-1^+^CD44^+^CD45.2^+^ CD8^+^ T cells from *Id2*^fl/fl^*Cd4*-Cre^+^ OT-I mice in tumors (Fig. 5E). In addition, the expression levels of stemness or progenitor markers, including Tcf1, Slamf6 and CXCR5, were decreased in adoptive CD8^+^ T cells from *Id2*^fl/fl^*Cd4*-Cre^+^ OT-I mice in both tumors and tdLNs (Fig. 5F). Furthermore, deletion of Id2 resulted in a decreased frequency of Tim-3^low^ cells among adoptively transferred PD-1^+^CD44^+^CD45.2^+^OT-I CD8^+^ T cells in tumors after T-cell transfer therapy (Fig. 5G). Id2 ablation led to a decreased proportion of Slamf6^+^ or CXCR5^+^ cells among adoptively transferred PD-1^+^CD44^+^Tim-3^low^CD45.2^+^OT-I CD8^+^ T cells in both tumors and tdLNs (Fig. 5H). Taken together, these results suggest that Id2 is necessary for the maintenance of stem-like populations of exhausted CD8^+^ T cells, presumably attributed to the retention of progenitor cells.

### Id2 promotes the generation of Slamf6^+^ Tex^prog^ cells and their conversion to Tex^term^ cells

**Fig. 6:**
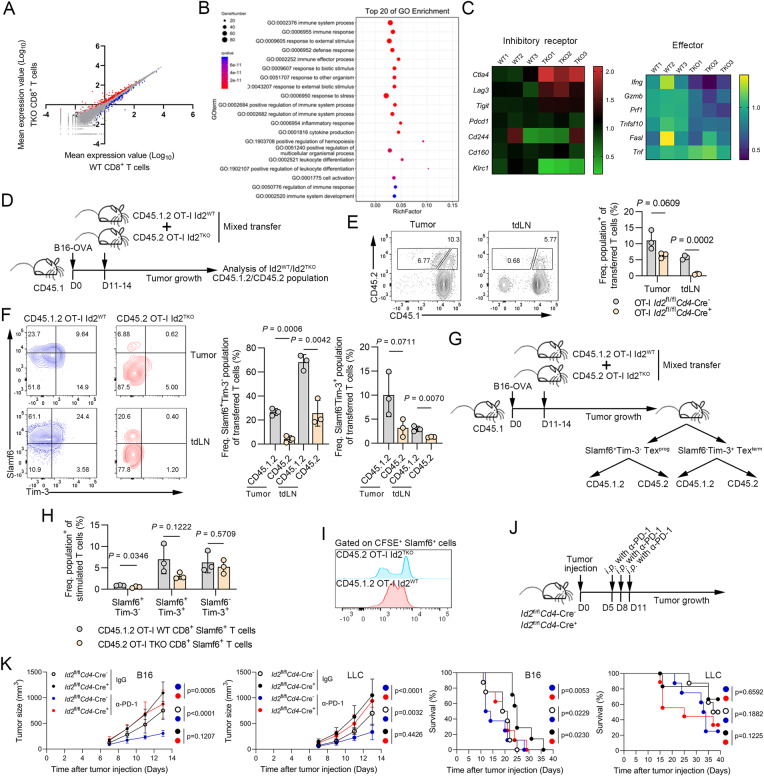
Id2 regulates the generation of Slamf6^+^ progenitor cells and their conversion to terminally exhausted CD8^+^ T cells in tumors. **A** Scatter plot comparing the global gene expression profiles of *Id2*^fl/fl^*Cd4*-Cre^-^ (WT) and *Id2*^fl/fl^*Cd4*-Cre^+^ (TKO) CD8^+^ T cells stimulated with anti-CD3 and anti-CD28 antibodies for 24 h. Transcripts with a log_10_ (fold change) of >0.5 and adjusted *p* value of <0.05 in TKO CD8^+^ T cells are shown in red (upregulated expression, 227 genes) or blue (downregulated expression, 113 genes). **B** Top 20 enriched GO terms in the comparison of *Id2*^fl/fl^*Cd4*-Cre^-^ CD8^+^ T cells and *Id2*^fl/fl^*Cd4*-Cre^+^ CD8^+^ T cells. **C** A heatmap of upregulated and downregulated immune checkpoint genes, including *Pdcd1, Ctla4, Lag3, Tigit, Cd244, Cd160* and *Klrc1*, in *Id2*^fl/fl^*Cd4*-Cre^+^ CD8^+^ T cells compared with *Id2*^fl/fl^*Cd4*-Cre^-^ CD8^+^ T cells. Each column shows data for one sample. A heatmap of upregulated and downregulated effector molecule-encoding genes, including *Ifng, Gzmb, Prf1, Tnfsf10, Fasl and Tnf*, in *Id2*^fl/fl^*Cd4*-Cre^+^ CD8^+^ T cells compared to *Id2*^fl/fl^*Cd4*-Cre^-^ CD8^+^ T cells. Each column shows data for one sample. **D** Schematic of the procedure for cotransfer of CD45.1.2 OT-I Id2^WT^ CD8^+^ T cells and CD45.2 OT-I Id2^TKO^ CD8^+^ T cells. **E** Flow cytometric analysis of the frequencies of cotransferred CD8^+^ T cells from CD45.1^.^2 OT-I Id2^WT^ mice and CD45.2 OT-I Id2^TKO^ mice in B16-OVA tumors and tdLNs. The data are presented as representative plots and summary graphs. **F** Representative flow cytometric plots of the frequencies of cotransferred Slamf6^+^Tim-3^-^ and Slamf6^-^Tim-3^+^ CD8^+^ T cells from CD45.1^.^2 OT-I Id2^WT^ mice and CD45.2 OT-I Id2^TKO^ mice in B16-OVA tumor and tdLNs. The data are presented as representative plots and summary graphs. **G** Schematic of the procedures for the in vitro conversion and CFSE proliferation assays using cotransferred CD45.1.2 OT-I Id2^WT^ CD8^+^ T cells and CD45.2 OT-I Id2^TKO^ CD8^+^ T cells isolated 15-18 days after B16-OVA tumor cell injection. **H** Summary graphs of the frequencies of cotransferred Slamf6^+^Tim-3^-^, Slamf6^+^Tim-3^+,^ and Slamf6^-^Tim-3^+^ CD8^+^ T cells from CD45.1^.^2 OT-I Id2^WT^ mice and CD45.2 OT-I Id2^TKO^ mice in B16-OVA tumors on Day 5. The data are presented with reference to Slamf6^+^ cells. **I** Quantification of cell divisions following in vitro stimulation (anti-CD3/CD28 antibodies with IL-2) of CFSE-labeled Slamf6^+^ CD45.1.2 OT-I Id2^WT^ CD8^+^ T cells and CD45.2 OT-I Id2^TKO^ CD8^+^ T cells isolated on Day 5. **J** Schematic of the procedure for anti-PD-1 treatment of *Id2*^fl/fl^*Cd4*-Cre^-^ and *Id2*^fl/fl^*Cd4*-Cre^+^ mice (intraperitoneal injection with an anti-PD-1 antibody or control antibody on Days 5, 8 and 11). **K** Tumor growth curves for *Id2*^fl/fl^*Cd4*-Cre^-^ mice and *Id2*^fl/fl^*Cd4*-Cre^+^ mice injected subcutaneously with 2 × 10^5^ B16-F10 melanoma cells or 2 × 10^6^ LLC lung cancer cells prior to intraperitoneal injection of the anti-PD-1 antibody on Days 5, 8 and 11. IgG, control antibody. Survival curves of *Id2*^fl/fl^*Cd4*-Cre^-^ mice and *Id2*^fl/fl^*Cd4*-Cre^+^ mice injected subcutaneously with 2 × 10^5^ B16-F10 melanoma cells or 2 × 10^6^ LLC lung cancer cells prior to intraperitoneal injection of the anti-PD-1 antibody on Days 5, 8 and 11. IgG, control antibody

To explore the Id2-mediated programs underlying CD8^+^ T-cell exhaustion, we performed RNA-seq using naïve CD8^+^ T cells activated in vitro to examine gene expression profiles. In comparison with *Id2*^fl/fl^*Cd4*-Cre^−^ CD8^+^ T cells, *Id2*^fl/fl^*Cd4*-Cre^+^ CD8^+^ T cells displayed upregulation of 227 genes and downregulation of 113 genes (Fig. 6A). Eleven of the top 20 enriched GO terms were related to immune responses or processes (e.g., immune system process (GO:0002376), immune response (GO:0006955), response to external stimulus (GO:0009605), etc.), which play an important role in T-cell exhaustion (Fig. 6B). A critical hallmark of exhausted T cells is upregulated and sustained expression of multiple inhibitory checkpoints [17]. Thus, we assessed the expression profiles of inhibitory receptors, including *Pdcd1, Ctla4, Lag3, Cd244, Tigit, Cd160* and *Klrc1*. Notably, upregulation of *Ctla4, Lag3* and *Tigit* was observed in *Id2*^fl/fl^*Cd4*-Cre^+^ CD8^+^ T cells compared to *Id2*^fl/fl^*Cd4*-Cre^−^ CD8^+^ T cells. Moreover, downregulation of effector molecules, including *Ifng, Gzmb* and *Prf1*, was observed in *Id2*^fl/fl^*Cd4*-Cre^+^ CD8^+^ T cells compared to *Id2*^fl/fl^*Cd4*-Cre^−^ CD8^+^ T cells (Fig. 6C). These findings support the role of Id2-mediated T-cell exhaustion.

Recent studies have shown that during chronic viral infections, exhausted CD8^+^ T cells develop into two major phenotypically and functionally distinct subpopulations: Slamf6^+^ Tex^prog^ cells and Tim-3^+^ Tex^term^ cells. The Tex^prog^ subpopulation is defined by a Slamf6^+^Tim-3^−^ signature and is characterized by polyfunctionality, prolonged persistence and a function as a reservoir for Tex^term^ cells. The Tex^term^ subpopulation is identified by a Slamf6^−^Tim-3^+^ signature, with greater cytotoxicity [8, 23, 24]. Next, we focused on whether Id2 can regulate the generation of Slamf6^+^ Tex^prog^ cells and their conversion to Tim-3^+^ Tex^term^ cells in tumors. Thus, to examine the cell-intrinsic role of Id2 in CD8^+^ T-cell exhaustion, we cotransferred congenically marked *Id2*^fl/fl^*Cd4*-Cre^+^ OT-I CD8^+^ T cells and *Id2*^fl/fl^*Cd4*-Cre^−^ OT-I CD8^+^ T cells into wild-type recipient mice challenged with B16-OVA cells (Fig. 6D and Supplementary Fig. 3A). A marginally decreased frequency was observed for adoptively transferred *Id2*^fl/fl^*Cd4*-Cre^+^ OT-I CD8^+^ T cells compared to *Id2*^fl/fl^*Cd4*-Cre^−^ OT-I CD8^+^ T cells in tumors, indicating that *Id2*^fl/fl^*Cd4*-Cre^−^ OT-I CD8^+^ T cells had a marked competitive advantage over *Id2*^fl/fl^*Cd4*-Cre^+^ OT-I CD8^+^ T cells. Consistently, a dramatic decrease in adoptively transferred *Id2*^fl/fl^*Cd4*-Cre^+^ OT-I CD8^+^ T cells was also detected in tdLNs (Fig. 6E). Next, we examined the impact of Id2 ablation on the generation of the Slamf6^+^ Tex^prog^ and Tim-3^+^ Tex^term^ subpopulations. We found that Id2 ablation decreased the frequencies of both Slamf6^+^ Tex^prog^ cells and Tim-3^+^ Tex^term^ cells in both tumors and tdLNs (Fig. 6F).

Next, we investigated whether the decreased frequencies of Slamf6^+^ cells and Tim-3^+^ cells were attributed to decreased conversion from Slamf6^+^ cells. We sorted antigen-experienced *Id2*^fl/fl^*Cd4*-Cre^−^ or *Id2*^fl/fl^*Cd4*-Cre^+^ Slamf6^+^ cells from B16-OVA tumors and restimulated them in vitro with anti-CD3 and anti-CD28 antibodies as well as IL-2 and found that compared to the *Id2*^fl/fl^*Cd4*-Cre^−^ subpopulation, the *Id2*^fl/fl^*Cd4*-Cre^+^ Slamf6^+^ subpopulation was attenuated to maintain the Slamf6^+^Tim-3^−^ subset, suggesting that Id2 inhibited the conversion of Slamf6^+^ Tex^prog^ cells to Tim-3^+^ Tex^term^ cells (Fig. 6H). Then, we examined the proliferative capacity following restimulation and found that *Id2*^fl/fl^*Cd4*-Cre^+^ Slamf6^+^ cells possessed an attenuated proliferative capacity (Fig. 6I). In addition, we assessed apoptosis in adoptive CD8^+^ T cells in recipient tumors and found no obvious difference between *Id2*^fl/fl^*Cd4*-Cre^+^ mice and their littermates (Supplementary Fig. 3B). Therefore, these results indicate that Id2 ablation decreases the conversion of Slamf6^+^ cells to Tim-3^+^ cells and decreases the proliferative capacity of Slamf6^+^ cells.

Given that Tex^prog^ cells but not Tex^term^ cells can respond to anti-PD-1 therapy, we treated tumor-bearing *Id2*^fl/fl^*Cd4*-Cre^−^ mice and *Id2*^fl/fl^*Cd4*-Cre^+^ mice with an anti-PD-1 antibody [8, 25] (Fig. 6J). Anti-PD-1 antibody administration dramatically inhibited tumor growth and prolonged the survival of tumor-bearing mice. However, no significant difference was observed in tumor growth between tumor-bearing *Id2*^fl/fl^*Cd4*-Cre^+^ mice treated with the anti-PD-1 antibody and those treated with control IgG, mainly due to the decreased population of Slamf6^+^ Tex^prog^ cells that can respond to PD-1 blockade (Fig. 6K). Collectively, these findings demonstrate that Id2 promotes the generation of Tex^prog^ cells and their conversion to Tex^term^ cells in tumors and restrains T-cell-mediated immune evasion.

### Id2 interacts with Tcf3 and disrupts the formation of the Tcf3-Tal1 complex

**Fig. 7:**
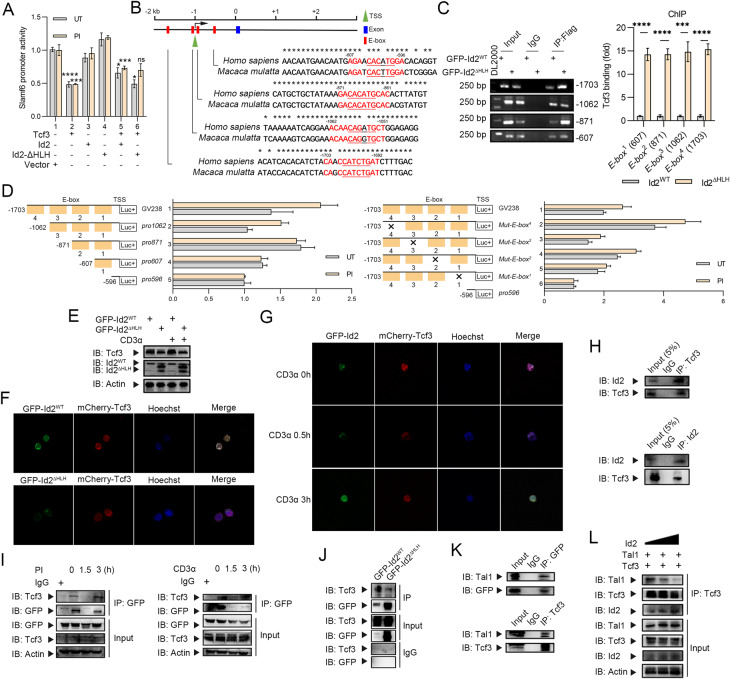
Id2 interacts with Tcf3 and disrupts the formation of the Tcf3-Tal1 complex. **A** Slamf6 promoter activity was evaluated by a dual luciferase reporter assay in HEK-293 cells. A plasmid containing the Slam6 promoter fused to the luciferase promoter gene was cotransfected with the Tcf3-GV219, Id2-GV219, or Id2-ΔHLH-GV219 expression plasmid or the empty GV219 vector alone and in combination. After 20 h, the cells were left untreated (UT) or treated with PMA plus ionomycin (PI) for 4 h. The transfection efficiency was controlled by cotransfection of a plasmid constitutively expressing Renilla luciferase. The results are presented as the ratio of firefly luciferase activity to Renilla luciferase activity. Asterisks from left to right: Group 1 (UT) vs. Group 2 (UT); Group 1 (PI) vs. Group 2 (PI); Group 2 (UT) vs. Group 5 (UT); Group 2 (PI) vs. Group 5 (PI); Group 5 (UT) vs. Group 6 (UT); Group 5 (PI) vs. Group 6 (PI). ns, not significant; *P < 0.05; ***P < 0.001; ****P < 0.0001. HLH, helix-loop-helix domain. **B** Structural schematics showing the structure of the *Slamf6* gene with the E-boxes (putative Tcf3-Tal1 binding elements). The human genomic sequence is shown as the base sequence on the *x*-axis. The four red boxes indicate the E-boxes, the blue box indicates the exon (only one exon within +3 kb) and the green triangle indicates the transcription start site (TSS). * indicates nucleotides conserved between *Homo sapiens* and *Macaca mulatta*. **C** Chromatin immunoprecipitation (**C**hIP) assay of Tcf3 binding to the E-boxes in the *Slamf6* promoter in GFP-Id2^WT^ and GFP-Id2^ΔHLH^ Jurkat cells. The purified DNA fragments were analyzed by gel electrophoresis and real-time quantitative PCR. ***P < 0.001; ****P < 0.0001. **D** The Tcf3 expression plasmid was cotransfected with a series of 5’-deletion mutants of the human *Slamf6* promoter or a series of four E-box mutants of the human Slamf6 promoter fused to the luciferase gene into HEK-293 cells. After 20 h, the cells were left untreated (UT) or treated with PMA plus ionomycin (PI) for 4 h. The transfection efficiency was controlled by cotransfection of a plasmid constitutively expressing Renilla luciferase. The results are presented as the ratio of firefly luciferase activity to Renilla luciferase activity. The full-length Slamf6-promoter luciferase construct (−2000/+71) was cloned and inserted into the GV238 vector. Slamf6 promoter subregions (−1703/+71, which contains all four E-boxes; −1062/+71, which contains three E-boxes; −871/+71, which contains two E-boxes; −607/+71, which contains only one E-box; and −596/+71, which contains none of the four E-boxes) were cloned and inserted into the GV238 vector. The four Slamf6 promoter E-boxes were disrupted by introducing mutations into their respective sequences using a mutagenesis kit according to the manufacturer’s instructions. Four mutated Slamf6 promoter sequences were also cloned and inserted into the GV238 vector: mutation of the fourth E-box mutation, 5’-CAAC**CA**TC**TG**AT-3’ → 5’-CAAC**TG**TC**GT**AT-3’; mutation of the third E-box mutation, 5’-ACAA**CA**GA**TG**CT-3’ → 5’-ACAA**TG**GA**GT**CT-3’; mutation of the second E-box mutation, 5’-GA**CA**CA**TG**CAC-3’ → 5’-GA**TG**CA**GT**CAC-3’; and mutation of the first E-box, 5’-AGAA**CA**CA**TG**GA-3’ → 5’-AGAA**TG**CA**GT**GA-3’. **E** Immunoblot analysis of Id2 and Tcf3 protein expression in GFP-Id2^WT^ and GFP-Id2^ΔHLH^ Jurkat cells treated with the anti-CD3 antibody. IB, immunoblot. **F**, **G** Confocal microscopy analysis of GFP-Id2 and mCherry-Tcf3 localization in GFP-Id2^WT^ and GFP-Id2^ΔHLH^ Jurkat cells with or without anti-CD3 antibody treatment. **H** Coimmunoprecipitation (IP) assay of the Id2-Tcf3 interaction in Jurkat cells. **I** Coimmunoprecipitation (IP) assay of the Id2-Tcf3 interaction in PI-stimulated HEK-293 cells and anti-CD3 antibody-stimulated Jurkat cells. **J** Coimmunoprecipitation (IP) assay of the Id2-Tcf3 interaction in GFP-Id2^WT^ and GFP-Id2^ΔHLH^ Jurkat cells. **K** Coimmunoprecipitation (IP) assay of the Id2-Tal1 interaction and Tal1-Tcf3 interaction in Jurkat cells. **L** Immunoblots showing the binding of Id2 and Tal1 to Tcf3. HEK-293 cells were transfected with a fixed concentration of Flag-Tcf3 and Tal1 and increasing concentrations of Id2

Next, we investigated how Id2 promotes the generation of Slamf6^+^ Tex^prog^ cells through transcriptional regulation. Id2 can form heterodimers with the transcription factor Tcf3 through its HLH dimerization domain [26]. In the presence of Id2, the Id2-Tcf3 interaction rather than the Tcf3-Tcf3 interaction dominates to orchestrate gene transcription [27]. First, we determined whether Id2-dependent Slamf6 expression is mediated through Tcf3. A Slamf6 (−2000/+71)/luciferase promoter construct was cotransfected with the Id2 (WT), Id2 (ΔHLH) or Tcf3 expression plasmid alone or in combination. We found that transfection of either Id2 (WT) or Id2 (ΔHLH) alone did not result in a change in *Slamf6* promoter activity. However, transfection of Tcf3 alone significantly inhibited *Slamf6* promoter activity, which was rescued by the addition of Id2 (WT) but not Id2 (ΔHLH), indicating that Id2-dependent Slamf6 induction was compromised by Tcf3 (Fig. 7A). Next, we assessed whether the *Slamf6* gene could be a direct target of Tcf3. Multiple putative Tcf3-occupied E-box (CANNTG) motifs were predicted by JASPAR in the human *Slamf6* locus, and we selected the top 4 evolutionarily conserved putative motifs (Fig. 7B). To directly elucidate the effects of Id2 on the binding of Tcf3 to the *Slamf6* promoter, chromatin immunoprecipitation (ChIP) assays were conducted in GFP-Id2^WT^ and GFP-Id2^ΔHLH^ Jurkat cells. We found that deletion of the HLH domain in Id2 dramatically enhanced the binding activity of Tcf3 to the *Slamf6* promoter (Fig. 7C). These findings indicate that Id2 may inhibit the binding activity of Tcf3 to the *Slamf6* promoter via heterodimerization with Tcf3 through its HLH domain.

Then, to map the Tcf3 binding sites in the *Slamf6* promoter, an expression plasmid containing full-length Tcf3 was cotransfected into HEK-293 cells with a series of 5’-deletion mutants of the human *Slamf6* promoter fused to the luciferase promoter gene. We observed no significant differences in the reporter activity among these five 5’-deletion mutants of the human *Slamf6* promoters, presumably owing to the long sequence between each mutation site, which may be occupied by other potential transcription factors. Thus, we generated point mutations in the four E-boxes in the *Slamf6* promoter instead of deletion mutations. Mutation of the fourth E-box (5’-CAAC**CA**TC**TG**AT-3’ → 5’-CAAC**TG**TC**GT**AT-3’) resulted in the maximum increase in reporter activity (Fig. 7D). These results indicate that Tcf3 preferentially binds to the fourth E-box of the *Slamf6* promoter.

Next, we determined the expression levels of Id2 and Tcf3 in Jurkat cells. Deletion of the HLH domain of Id2 did not alter the expression level of Tcf3 (Fig. 7E). In addition, Id2 colocalization with Tcf3 was observed in Jurkat cells and was compromised by deletion of the HLH domain (Fig. 7F and Supplementary Fig. 4A). With prolongation of anti-CD3 antibody treatment, the colocalization and interaction of Id2 and Tcf3 were slightly decreased and then restored, as demonstrated by confocal microscopy (Fig. 7G and Supplementary Fig. 4B) and coimmunoprecipitation (Co-IP) assays (Fig. 7H, I; Supplementary Fig. 4C). Further Co-IP assays confirmed that deletion of the HLH domain of Id2 attenuated the association of Id2 with Tcf3 (Fig. 7J). Of note, the Tcf3-occupied sites in the *Slamf6* promoter were also often occupied by another transcription factor, Tal1, as predicted by JASPAR. We found that Id2 could interact with Tal1 and that Tcf3 could also interact with Tal1 (Fig. 7K). When Tcf3 was immunoprecipitated after increased expression of Id2, we observed a dose-dependent reduction in the Tal1 abundance in the Tcf3 coimmunoprecipitate (Fig. 7L). Therefore, these results suggest that the Id2-Tcf3 interaction disrupts the formation of the Tcf3-Tal1 complex, thereby promoting Slamf6 induction.

### HLH-deficient Id2 facilitates the formation of the Tcf3-Tal1 complex to decrease chromatin accessibility and promote Tex^prog^-to-Tex^term^ conversion by recruiting LSD1

**Fig. 8:**
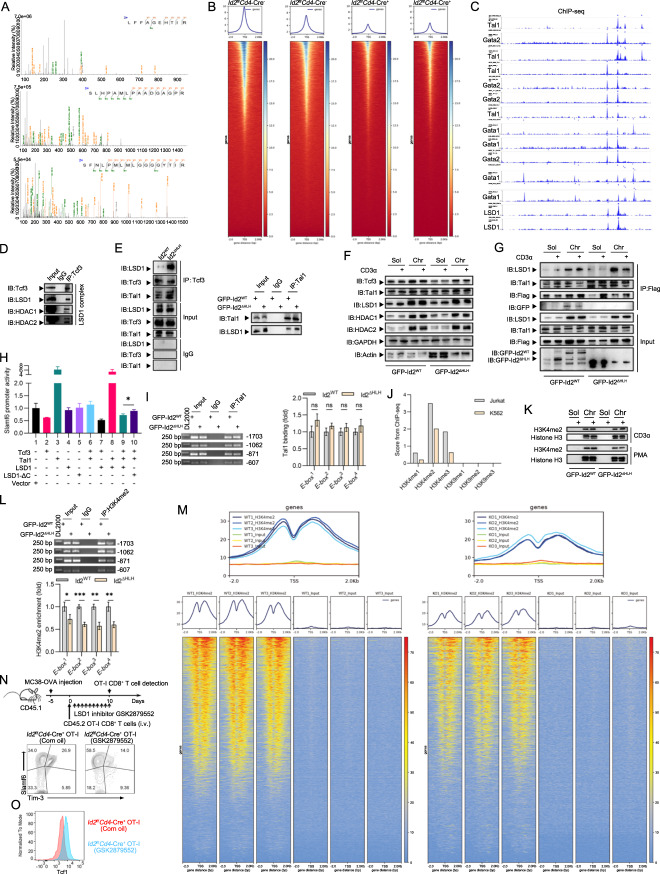
Id2^ΔHLH^ allows the Tcf3-Tal1 complex to recruit LSD1 to alter chromatin accessibility during the Tex^prog^-to-Tex^term^ conversion. **A** LC/MS analysis of Tcf3-associated proteins in Jurkat cells after the Tcf3 pull-down assay. From top to bottom: LSD1, Tal1 and HDAC1. **B** ATAC-seq analysis of splenic *Id2*^fl/fl^*Cd4*-Cre^-^ and *Id2*^fl/fl^*Cd4*-Cre^+^ CD8^+^ T cells stimulated with anti-CD3/CD28 antibodies for 24 h. **C** Sites of Tal1, LSD1, Gata1 and Gata2 occupancy at the chromosomal locus of Slamf6 were obtained from Cistrome DB, a ChIP-seq database. **D** The LSD1 complex associated with Tcf3 was pulled down from Jurkat cells. **E** Coimmunoprecipitation (IP) assay of the Tal1-Tcf3 interaction, LSD1-Tcf3 interaction and Tal1-LSD1 interaction in GFP-Id2^WT^ and GFP-Id2^ΔHLH^ Jurkat cells. **F** Immunoblot analysis of cytosolic and nuclear extracts from GFP-Id2^WT^ and GFP-Id2^ΔHLH^ Jurkat cells treated with or without the anti-CD3 antibody. Markers for the soluble fraction: GAPDH and actin; markers for the chromatin fraction: HDAC1 and HDAC2. **G** Coimmunoprecipitation (IP) assay of the Tal1-Tcf3 interaction and LSD1-Tcf3 interaction in the chromatin and soluble fractions extracted from GFP-Id2^WT^ and GFP-Id2^ΔHLH^ Jurkat cells with or without anti-CD3 antibody stimulation. **H** Slamf6 promoter activity was evaluated by a dual luciferase reporter assay in HEK-293 cells. A plasmid containing the Slam6 promoter fused to the luciferase promoter gene was cotransfected with either the Tcf3-GV712, Tal1-GV712, LSD1 (WT)-GV712 or LSD1 (ΔC)-GV712 expression plasmid or the empty GV712 vector alone or in combination. The transfection efficiency was controlled by cotransfection of a plasmid constitutively expressing Renilla luciferase. The results are presented as the ratio of firefly luciferase activity to Renilla luciferase activity. C, C-terminal amine oxidase domain. *P < 0.05. **I** ChIP assay of Tal1 binding to E-boxes in the *Slamf6* promoter in GFP-Id2^WT^ and GFP-Id2^ΔHLH^ Jurkat cells. The purified DNA fragments were analyzed by gel electrophoresis and real-time quantitative PCR. ns, not significant. **J** H3 lysine 4 (H3K4) and H3 lysine 9 (H3K9) methylation scores of the *Slamf6* gene in Jurkat and K562 cells. The data were derived from Cistrome DB. **K** Immunoblot analysis of histone H3 (lysine 4) dimethylation in GFP-Id2^WT^ and GFP-Id2^ΔHLH^ Jurkat cells stimulated with the anti-CD3 antibody or with PMA plus ionomycin. **L** ChIP assay of H3K4me2 binding to E-boxes in the *Slamf6* promoter in GFP-Id2^WT^ and GFP-Id2^ΔHLH^ Jurkat cells. The purified DNA fragments were analyzed by gel electrophoresis and real-time quantitative PCR. * *P* < 0.05; ** *P* < 0.01; *** *P* < 0.001. **M** Global H3K4me2 level in antigen-experienced OT-I CD8^+^ T cells in B16-OVA tumors. WT, *Id2*^fl/fl^*Cd4-*Cre^-^; KO, *Id2*^fl/fl^*Cd4*-Cre^+^. **N** Experimental design for daily treatment with the chemical LSD1 inhibitor GSK2879552 to rescue the Id2 deletion phenotype. Assessment of Tex^prog^ and Tex^term^ cells among adoptive *Id2*^fl/fl^*Cd4*-Cre^+^ OT-I CD8^+^ T cells in MC38-OVA tumors from mice treated with corn oil or GSK2879552. **O** Assessment of Tcf1 in adoptive *Id2*^fl/fl^*Cd4*-Cre^+^ OT-I CD8^+^ T cells in MC38-OVA tumors from mice treated with corn oil or GSK2879552

To illustrate the Tcf3-mediated transcriptional programs of Slamf6, we performed immunoprecipitation followed by mass spectrometry (MS) to identify the Tcf3 interactome. In our MS analysis, we focused mainly on the clusters related to nucleotide-binding processes, which might play an important role in epigenetic modification. We found that the components of the LSD1 complex (LSD1, HDAC1, etc.) were pulled down with Tcf3 (Fig. 8A). The core LSD1 complex is composed of four subunits: LSD1, HDAC1, HDAC2, and CoREST [28, 29]. HDAC1, HDAC2 and CoREST can enhance the binding of LSD1 to hypoacetylated nucleosomes [29–31]. Based on these data, we hypothesized that Id2 might affect the nucleosome configuration and chromatin accessibility via LSD1-mediated histone methylation. Thus, we performed assay for transposase-accessible chromatin sequencing (ATAC-seq) to map open chromatin regions in *Id2*^fl/fl^*Cd4*-Cre^+^ and *Id2*^fl/fl^*Cd4*-Cre^−^ CD8^+^ T cells and found that deletion of Id2 globally decreased chromatin accessibility at gene loci, including at the promoter of *Slamf6* (Fig. 8B; Supplementary Fig. 5A). By searching the Cistrome DB ToolKit and WashU Browser, we identified that the putative binding sites of LSD1, Tal1 and Gata1/2 (often associated with Tal1) were highly consistent in the *Slamf6* gene (Fig. 8C). Thus, we speculated that Tcf3 can bind Tal1 and form the Tcf3-Tal1 transcriptional regulatory complex, subsequently recruiting the LSD1 complex to demethylate H3K4 in the *Slamf6* gene. Co-IP assays confirmed that Tcf3 interacted with the subunits of the LSD1 complex LSD1, HDAC1 and HDAC2 (Fig. 8D). Next, we examined whether Id2 affects the association of Tcf3 with Tal1 or LSD1. We found that HLH-deleted Id2 resulted in enhanced Tcf3-Tal1, Tcf3-LSD1, and Tal1-LSD1 binding (Fig. 8E). Considering that transcription factors may play roles through translocation from the cytoplasm to the nucleus, we then performed a nuclear-cytoplasmic separation assay. We found that HLH deletion in Id2 did not result in significant changes in the levels of cytoplasmic and nuclear Tcf3, Tal1 and LSD1 (Fig. 8F). However, we found that HLH deletion in Id2 resulted in enhanced binding of LSD1 and Tal1 to Tcf3, thereby forming the Tcf3/Tal1/LSD1 complex to modify the methylation status of the *Slamf6* gene (Fig. 8G). Moreover, we identified the domains involved in the Tcf3-LSD1 interaction. Tcf3 and its truncation mutants were inserted into Flag-tagged constructs (Supplementary Fig. 5B). The Co-IP assay confirmed that LSD1 mainly interacted with the DES domain and AD3 domain of Tcf3 (Supplementary Fig. 5C). Thus, we found that Id2 interacted with Tcf3 and disrupted the formation of the Tcf3-Tal1 complex, which binds to the *Slamf6* gene. Then, we asked whether Tal1 can also inhibit the transcription of Slamf6. We performed a dual luciferase reporter assay and unexpectedly found that transfection of Tal1 alone dramatically increased the reporter activity of *Slamf6*, an effect opposite that of Tcf3. Moreover, cotransfection of Tcf3 and Tal1 compromised the Tal1-mediated induction of *Slamf6* reporter activity. In addition, transfection of a C-terminal-deleted LSD1 mutant restored *Slamf6* reporter activity, indicating that LSD1 interacts with Tcf3 or Tal1 via its C-terminal domain (Fig. 8H). Next, we performed ChIP assays and found that HLH deletion in Id2 did not result in a significant change in the binding activity of Tal1 to the *Slamf6* promoter, thereby suggesting that Tal1 does not play an important role in the regulation of Slamf6 transcription (Fig. 8I). Furthermore, we identified the specific modification type mediated by LSD1 in the *Slamf6* promoter. Analysis of Cistrome DB showed that H3K4me2 is the major modification on the *Slamf6* gene, suggesting a role for LSD1 (Fig. 8J). Cellular fractionation assays showed that HLH deletion in Id2 led to a decrease in H3K4me2, especially with PMA stimulation (Fig. 8K). Furthermore, we performed ChIP assays and found that deletion of the HLH domain of Id2 significantly decreased the H3K4me2 level of the four E-boxes in the *Slamf6* promoter (Fig. 8L). We further performed ChIP-seq analysis to investigate the H3K4me2 level in adoptively transferred *Id2*^fl/fl^*Cd4*-Cre^+^ and *Id2*^fl/fl^*Cd4*-Cre^−^ CD8^+^ T cells in the B16-OVA model. Consistent with the ATAC-seq results, deletion of the HLH domain of Id2 resulted in global attenuation of H3K4me2 (Fig. 8M). Finally, we investigated whether a chemical LSD1 inhibitor GSK2879552, which can act on LSD1 in T cells and exert an effect similar to that of T-cell-specific LSD1 depletion [32], can mimic the Id2 knockout phenotype. We found that GSK2879552 restored the Id2 knockout phenotype. For instance, GSK2879552 increased the abundance of Slamf6^+^Tim-3^−^ Tex^prog^ cells in MC38-OVA tumor-bearing recipients in the adoptive transfer models (Fig. 8N). GSK2879552 also rectified the decrease in the level of Tcf1 in *Id2*^fl/fl^*Cd4*-Cre^+^ OT-I CD8^+^ T cells (Fig. 8O). In addition, GSK2879552 rectified the decreases in the levels of IFN-γ and CXCR5 and the increases in the levels of inhibitory receptors such as CD244 and PD-1 (Supplementary Fig. 5D). Taken together, these results demonstrate that the Tcf3-Tal1 complex dominates in the absence of the Id2 HLH domain and recruits LSD1 to alter chromatin accessibility at the *Slamf6* promoter during the Tex^prog^-to-Tex^term^ conversion in tumor immune evasion.

## Discussion

Delineating the mechanisms that modulate CD8^+^ T-cell exhaustion in chronic infections and cancer is critical for suppressing or circumventing immune evasion and enhancing the efficacy of cancer immunotherapy [17, 33, 34]. The mechanisms that orchestrate the generation of the Slamf6^+^ Tex^prog^ subpopulation and the balance between the Slamf6^+^ Tex^prog^ and Tim-3^+^ Tex^term^ subpopulations remain unclear. Here, we showed that Id2 transcriptionally and epigenetically regulates the generation of Slamf6^+^ Tex^prog^ cells and their conversion to Tex^term^ cells and thus restrains immune evasion. We found that genetic deletion of Id2 dampens CD8^+^ T-cell-mediated immune responses, suppresses PD-1 blockade and increases tumor susceptibility in mice. Moreover, Id2 sustains the maintenance of stem cell-like CD8^+^ T cells after T-cell therapy. Mechanistically, Id2 binds and disrupts the assembly of the Tcf3-Tal1 transcriptional regulatory complex, inhibits the function of Tcf3 in recruiting and binding LSD1, increases the abundance of the permissive H3K4me2 mark on the Tcf3-occupied E-boxes in the *Slamf6* promoter, and thus epigenetically regulates the generation of Slamf6^+^ Tex^prog^ cells (Supplementary Fig. 6). Collectively, our study demonstrates that Id2-mediated transcriptional and epigenetic modifications play a crucial role in hierarchical T-cell exhaustion, and the mechanistic insights gained may have implications for therapeutic intervention with tumor immune evasion.

Our work identifies Id2 as a novel transcriptional regulator of CD8^+^ T-cell exhaustion in cancer. A previous study reported that Id2 was prominently upregulated in late CD8^+^ effector T cells and that its expression was maintained in memory cells in a murine model of Listeria infection [35]. Likewise, we found increased expression of Id2 in tumor-infiltrating CD8^+^ T cells from patients with cancer, indicating that Id2 may be upregulated in activated T cells. Deletion of Id2 in CD8^+^ T cells resulted in diminished clearance of Listeria after infection [35]. Consistently, in this study, we found that Id2 ablation in CD8^+^ T cells promoted tumor development by perturbing immune responses, emphasizing the critical role of Id2 in host defense during infection and in cancer. Using an adoptive cell transfer model, we found that Id2 ablation interferes with the expansion and cytokine production of therapeutic CD8^+^ T cells in tumors. Regarding the impact on memory and effector phenotypes, previous studies have shown that Id2-knockout mice infected with Listeria lack the effector memory subset [35], consistent with our findings in tumors. Notably, a marginal but nonsignificant decrease in the central memory subset was observed in adoptive *Id2*^fl/fl^*Cd4*-Cre^+^ OT-I CD8^+^ T cells, consistent with a previous study that reported poor enrichment of central memory signatures in progenitor exhausted CD8^+^ T cells [8]. Moreover, we found that the expression levels of stemness and progenitor markers, including Tcf1, Slamf6 and CXCR5, were also decreased in adoptively transferred CD8^+^ T cells from *Id2*^fl/fl^*Cd4*-Cre^+^ OT-I mice in both tumors and tdLNs, indicating that Id2 sustains the maintenance of stem cell-like CD8^+^ T cells after T-cell therapy.

Kinases and transcription factors have been reported to regulate the generation and conversion of exhausted CD8^+^ T-cell subpopulations during LCMV infection and in cancers [23, 24, 36]. Id2 has been shown to regulate the production of different CD8^+^ T-cell subsets [37–44]. In this study, we found that Id2 regulates the generation of Slamf6^+^ Tex^prog^ cells and the Tex^prog^-to-Tex^term^ conversion in cancer. We found decreases in the frequencies of both Slamf6^+^ Tex^prog^ cells and Tim-3^+^ Tex^term^ cells. The absence of Id2 in CD8^+^ T cells can promote cell death [35], which was rarely changed in tumors. Thus, we mainly focused on the following presumptions: (1) decreased generation of Slamf6^+^ cells; (2) decreased conversion of Slamf6^+^ cells; and (3) decreased proliferation of either subpopulation. We found attenuated maintenance of Slamf6^+^Tim-3^−^ subsets among OT-I Id2^TKO^ Slamf6^+^ cells compared to OT-I Id2^WT^ cells, suggesting that Id2 inhibits the conversion of Slamf6^+^ Tex^prog^ cells to Tim-3^+^ Tex^term^ cells. However, no difference in conversion was observed for restimulated Tim-3^+^ cells. Then, we examined the proliferative capacity following restimulation and found that OT-I Id2^TKO^ Slamf6^+^ cells possessed a reduced proliferative capacity. Therefore, these results suggest that Id2 ablation affects the conversion of Slamf6^+^ Tex^prog^ cells to Tim-3^+^ Tex^term^ cells and decreases the proliferative capacity of Slamf6^+^ Tex^prog^ cells. Considering that Tex^prog^ cells but not terminally exhausted T cells can respond to anti-PD-1 therapy [8], we treated tumor-bearing mice with PD-1 blockade and found that Id2 deletion impaired the generation or maintenance of Slamf6^+^ Tex^prog^ cells that can respond to PD-1 blockade. Taken together, these findings demonstrate that Id2 regulates the Tex^prog^-to-Tex^term^ conversion in tumors and restrains T-cell-mediated immune evasion.

CD8^+^ T cell exhaustion is accompanied by transcriptional and epigenetic reprogramming [45, 46]. Several groups have reported T-cell exhaustion as an epigenetic continuum [47–49]. We found that Id2 preferentially binds and disrupts the assembly of the Tcf3-Tal1 transcriptional regulatory complex through its HLH domain and modulates chromatin accessibility at the *Slamf6* promoter. Vital to epigenetic regulation is the diverse repertoire of covalent modifications on histones that dynamically orchestrate chromatin accessibility. HLH deletion in Id2 allows Tcf3 to recruit and bind the histone lysine demethylase LSD1, consistent with the results of a previous biochemical analysis [50]. LSD1 diminishes the abundance of the permissive H3K4me2 mark on the corresponding E-boxes of the *Slamf6* promoter and thus results in epigenetic reprogramming in Slamf6^+^ Tex^prog^ cells. The LSD1 inhibitor GSK2879552 can rescue the Id2 knockout phenotype. A study reported that genetic downregulation of Id3, a cognate regulator of Id2, can repress CAR-T-cell exhaustion and improve the efficacy of CAR T-cell therapy in solid tumors [51]. Based on our findings, targeting Id2 and its interactome is expected to enhance the antitumor immune activity of adoptive T cells.

Overall, in this study, we reveal that Id2 restrains immune evasion by disrupting the formation of the Tcf3-Tal1 transcriptional regulatory complex and its recruitment of the histone lysine demethylase LSD1, thereby increasing the abundance of the permissive H3K4me2 mark during hierarchical CD8^+^ T-cell exhaustion in cancer. Our study demonstrates that Id2-mediated transcriptional and epigenetic modifications play a crucial role in hierarchical T-cell exhaustion, and the mechanistic insights gained may have implications for therapeutic intervention with tumor immune evasion.

## Materials and methods

### Mice

*Id2*^fl/fl^ (CD45.2) mice and *Cd4*^Cre^ transgenic (CD45.2) mice were gifts from Dr. Yuzhang Wu, Third Military Medical University. *Id2*^fl/fl^ mice were crossed with *Cd4*^Cre^ transgenic mice to generate age-matched *Id2*^fl/fl^*Cd4*-Cre^−^ (Id2^WT^) and *Id2*^fl/fl^*Cd4*-Cre^+^ (Id2^TKO^) mice. OT-I (C57BL/6-Tg(TcraTcrb)1100Mjb/J) TCR-transgenic (CD45.2) mice, Rag1^−/−^ mice and CD45.1 (B6.SJL-Ptprca Pepcb/BoyJ) mice were fed in our animal center. OT-I mice were crossed with Id2^TKO^ mice to generate *Id2*^fl/fl^*Cd4*-Cre^+^ OT-I (OT-I Id2^TKO^) mice. *Id2*^fl/fl^*Cd4*-Cre^−^ OT-I (OT-I Id2^WT^) mice were crossed with B6.SJL mice to generate CD45.1.2 OT-I Id2^WT^ mice. For animal experiments, 6- to 10-week-old sex-matched mice were used. Mice were maintained on a 12 h light/dark cycle (light from 8 am to 8 pm and dark from 8 pm to 8 am) and were housed and maintained at stable room temperature under specific pathogen-free (SPF) conditions.

### Cell lines

B16-F10 melanoma cells, B16-OVA (OVA-expressing B16-F10) melanoma cells, LLC lung cancer cells, LLC-OVA (OVA-expressing LLC) lung cancer cells, Hepa1-6 hepatoma cells, Hepa1-6-OVA (OVA-expressing Hepa1-6) hepatoma cells, HEK-293 cells, and Jurkat cells were cultured in RPMI 1640 medium supplemented with 10% fetal bovine serum at 37 °C in a humidified atmosphere (5% CO_2_).

### Tumor models

Mice were injected subcutaneously in the flank with 2 × 10^5^ melanoma cells, 2 × 10^6^ lung cancer cells or 2 × 10^6^ hepatoma cells. The challenged mice were monitored for tumor size using a caliper every 2 d after tumors became palpable. For ICB therapy, mice were injected intraperitoneally with 100 μg of a hamster monoclonal antibody against PD-1 (clone RMP1-14, BioXCell) or hamster polyclonal IgG (BioXCell) on Days 5, 8 and 11 following tumor cell injection. Tumor volume was determined by the ellipsoid volume formula (1/2×*D*×*d*^2^), where *D* is the longest diameter and *d* is the shortest diameter. For LSD1 inhibitor administration, mice were injected intraperitoneally with GSK2879552 (1.5 mg/kg body weight) or corn oil daily after tumor cell injection.

### Diethylnitrosamine (DEN)-induced liver tumorigenesis

*Id2*^fl/fl^*Cd4*-Cre^−^ mice and *Id2*^fl/fl^*Cd4*-Cre^+^ mice were injected intraperitoneally with diethylnitrosamine (DEN) (25 mg/kg body weight; Sigma‒Aldrich) on postnatal day 14. The mice were fed at stable room temperature under specific pathogen-free (SPF) conditions and sacrificed after 40 weeks.

### Flow cytometry

Cells were stained with the indicated surface marker-specific antibodies in PBS containing 2% fetal bovine serum at 4 °C for 20 min. For intracellular cytokine staining, cells were stimulated with phorbol 12-myristate 13-acetate (PMA) and ionomycin for 6 h. After stimulation, cells were stained with the surface marker-specific antibodies at 4 °C for 20 min, fixed and permeabilized at 4 °C for 20 min with the Fixation/Permeabilization Solution Kit (BD Pharmingen), and then stained with the indicated intracellular antibodies at 4 °C for 20 min. Transcription factor staining was performed after surface staining as per the manufacturer’s instructions. Flow cytometry data were collected on a BD LSRFortessa instrument and analyzed with FlowJo software.

### Purification and propagation of primary murine T cells

Murine spleens were excised, mechanically minced and filtered to generate a single-cell suspension. CD8^+^ T cells were then isolated by flow sorting (BD FACSAria III). Isolated primary CD8^+^ T cells were cultured in murine T-cell medium containing RPMI 1640 medium ( + L-glutamine) supplemented with fetal bovine serum (10%), sodium pyruvate (5 mM), HEPES (5 mM), glutamine (2 mM), Pen/Strep (50 µg/mL), NEAA (5 mM) and β-mercaptoethanol (50 μM). Cells were then maintained at 37 °C in a humidified atmosphere (5% CO_2_).

### Tumor-infiltrating lymphocyte isolation

Tumors were excised, mechanically minced, incubated in collagenase solutions for 20 min at 37 °C, and then filtered to generate a single-cell suspension. After filtration, the cells were subjected to antibody staining at 4 °C for 20 min in RPMI 1640 medium and sorted by flow cytometry (BD FACSAria III).

### Quantification of DEN-induced tumor burden

Liver lobes were separately examined from every side. Tumor foci more than 1 mm in diameter were counted and measured [52].

### H&E staining

Liver specimens from DEN-induced mice were fixed with 10% neutral buffered formalin prior to paraffin embedding, sectioning and H&E staining. Images of histological staining were obtained using an Olympus microscope. H&E staining was assessed for indicators of malignancy.

### Adoptive T-cell transfer

Splenic T cells were sorted using flow cytometry (BD FACSAria III) and activated by incubation with anti-CD3 and anti-CD28 antibodies. Adoptive T-cell transfer was conducted in tumor-bearing recipient mice using intravenous injection and evaluated by monitoring tumor growth and analyzing the frequencies, functions and stemness of adoptive T cells in tumors and tdLNs. For mixed transfer to Rag1^−/−^ mice, tumor-bearing recipient mice were injected intravenously with 2 × 10^6^ *Id2*^fl/fl^*Cd4*-Cre^−^ or *Id2*^fl/fl^*Cd4*-Cre^+^ CD8^+^ T cells along with 2 × 10^6^ *Id2*^fl/fl^*Cd4*-Cre^−^ CD4^+^ T cells. For individual transfer to B6.SJL mice, tumor-bearing recipient mice were injected intravenously with 2 × 10^6^ OT-I *Id2*^fl/fl^*Cd4*-Cre^−^ or OT-I *Id2*^fl/fl^*Cd4*-Cre^+^ CD8^+^ T cells. For cotransfer to B6.SJL mice, tumor-bearing recipient mice were injected intravenously with CD45.1.2 OT-I Id2^WT^ CD8^+^ T cells and CD45.2 OT-I Id2^TKO^ CD8^+^ T cells (2 × 10^6^ cells of each type). The transferred T cells in tumors and tdLNs were analyzed by flow cytometry at the indicated time points based on the expression of the CD45.1 and CD45.2 congenic markers.

### In vitro T-cell differentiation assay using naïve CD8^+^ T cells

Naïve splenic CD8^+^ T cells were isolated from *Id2*^fl/fl^*Cd4*-Cre^−^ mice and *Id2*^fl/fl^*Cd4*-Cre^+^ mice by flow cytometry. The naïve CD8^+^ T cells were then stimulated by plate-bound anti-CD3 and anti-CD28 antibodies, with IL-2 (200 µ/mL) supplementation.

### In vitro cytotoxicity assays using tumor-specific CD8^+^ T cells

All in vitro cytotoxicity assays were conducted in murine T-cell medium (without addition of IL-2). Cells were cocultured in 96-well polystyrene microplates (Thermo Fisher) coated with collagen I (5 μg/cm^2^) according to the manufacturer’s instructions. Murine Hepa1-6 hepatoma cells expressing the OVA_257-264_ (SIINFEKL) peptide and GFP (Hepa1-6^GFP^-SIINFEKL) were pretreated with 5 ng/mL IFN-γ to induce upregulation of surface MHC class I protein expression [53]. Hepa1-6^GFP^-SIINFEKL cells were then cocultured with murine CD8^+^ T cells isolated from *Id2*^fl/fl^*Cd4*-Cre^−^ OT-I mice or *Id2*^fl/fl^*Cd4*-Cre^+^ OT-I mice that recognized SIINFEKL peptides presented by H2-K^b^ molecules. Murine CD8^+^ T cells were added at increasing effector-to-target ratios. The total number of surviving tumor cells was quantified 48 h after coculture using image cytometry.

### In vitro conversion assays using antigen-experienced Slamf6^+^ CD8^+^ T cells

B16-OVA tumor-bearing B6.SJL mice were injected intravenously with CD45.1.2 OT-I Id2^WT^ CD8^+^ T cells and CD45.2 OT-I Id2^TKO^ CD8^+^ T cells (2 × 10^6^ cells of each type). The transferred CD8^+^ T cells in tumors were isolated from recipient mice, and flow sorting for Slamf6^+^ CD8^+^ T cells was conducted at the indicated time points based on the expression of the CD45.1 and CD45.2 congenic markers. The sorted Slamf6^+^ CD8^+^ T cells were activated by plate-bound anti-CD3 and anti-CD28 antibodies, with IL-2 (200 µ/mL) supplementation. Cells were then in vitro conversed and detected by flow cytometry (BD LSRFortessa).

### In vitro CFSE proliferation assays using antigen-experienced Slamf6^+^ CD8^+^ T cells

B16-OVA tumor-bearing B6.SJL mice were injected intravenously with CD45.1.2 OT-I Id2^WT^ CD8^+^ T cells and CD45.2 OT-I Id2^TKO^ CD8^+^ T cells (2 × 10^6^ cells of each type). The transferred CD8^+^ T cells in tumors were isolated from recipient mice and sorted by flow cytometry for Slamf6^+^ CD8^+^ T cells at the indicated time points based on the CD45.1 and CD45.2 congenic markers. The sorted Slamf6^+^ CD8^+^ T cells were labeled with CFSE for 15 min at 37 °C. These cells were activated by plate-bound anti-CD3 and anti-CD28 antibodies, with IL-2 (200 µ/mL) supplementation, for 24 h. Cell proliferation was assessed by flow cytometry (BD LSRFortessa).

### RNA-seq analysis

*Id2*^fl/fl^*Cd4*-Cre^−^ CD8^+^ T cells and *Id2*^fl/fl^*Cd4*-Cre^+^ CD8^+^ T cells were isolated from 6-week-old *Id2*^fl/fl^*Cd4*-Cre^−^ mice (*n* = 3) and *Id2*^fl/fl^*Cd4*-Cre^+^ mice (*n* = 3). Naïve CD8^+^ T cells were sorted by flow cytometry and stimulated with anti-CD3 and anti-CD28 antibodies for 24 h, and total RNA isolation and RNA sequencing were then performed. Differential RNA expression analysis between different pairs of groups was performed with DESeq2 software. Genes with a false discovery rate (FDR) of <0.05 and an absolute fold change in expression of ≥2 were considered differentially expressed genes (DEGs). Through GO enrichment analysis, all GO terms that were significantly enriched in the DEGs in comparison with the genomic background were identified, and the DEGs that corresponded to the biological functions were filtered. All DEGs were mapped to GO terms in the Gene Ontology database (http://www.geneontology.org/), and gene counts were calculated for every term, and GO terms significantly enriched in the DEGs compared with the genomic background were defined by a hypergeometric test.

### Apoptosis assay

Adoptive *Id2*^fl/fl^*Cd4*-Cre^−^ and *Id2*^fl/fl^*Cd4*-Cre^+^ OT-I CD8^+^ T cells in B16-OVA tumors were stained with Annexin V and PI to assess apoptosis.

### Bioinformatic analysis

Data for the distribution of *Id2* expression in different immune cell types across multiple datasets were acquired from the TISCH database (http://tisch.comp-genomics.org/), which is a database of scRNA-seq data focusing on the tumor microenvironment across cancer types at the single-cell level. RNA-seq data for LUAD, SKCM and LIHC samples were acquired from the TCGA database, and quantification of immune cell populations in cancer patients with varying *Id2* expression levels was determined by the CIBERSORT tool [13]. Correlation analysis between the expression of *Id2* and inhibitory receptors (*Pdcd1*, *Ctla4*, *Cd244*, *Lag3*, *Tigit, Cd160 and Klrc1*) in adrenocortical carcinoma was conducted by TIMER2.0 (http://timer.cistrome.org/) [54]. Kaplan‒Meier plots of overall survival in patients stratified by both the estimated infiltration level of CD8^+^ T cells and the *Id2* expression level in the SKCM and SKCM-metastasis subgroups were also acquired from TIMER2.0. The occupancy of Tcf3 and Tal1 in the promoter of Slamf6 was predicted by JASPAR (https://jaspar.genereg.net/) [55]. Histone modification data for Slamf6 were obtained from Cistrome DB [56, 57].

### Plasmids, antibodies and reagents

The full-length Id2 (WT), full-length Tcf3 (for Fig. 7A), and HLH-deleted Id2 (Id2-ΔHLH) sequences were cloned and inserted into the GV219 vector. The full-length Slamf6-promoter luciferase construct (−2000/ + 71) was inserted into the GV238 vector. Slamf6 promoter subregions (−1703/ + 71, which contains all four E-boxes; −1062/ + 71, which contains three E-boxes; −871/ + 71, which contains two E-boxes; −607/ + 71, which contains only one E-box; and −596/ + 71, which contains none of the four E-boxes) were cloned and inserted into the GV238 vector. The four Slamf6 promoter E-boxes were disrupted by introducing mutations into their respective sequences using a mutagenesis kit according to the manufacturer’s instructions. Four mutated Slamf6 promoter sequences were also cloned and inserted into the GV238 vector: mutation of the fourth E-box, 5’-CAAC**CA**TC**TG**AT-3’ → 5’-CAAC**TG**TC**GT**AT-3’; mutation of the third E-box, 5’-ACAA**CA**GA**TG**CT-3’ → 5’-ACAA**TG**GA**GT**CT-3’; mutation of the second E-box, 5’-GA**CA**CA**TG**CAC-3’ → 5’-GA**TG**CA**GT**CAC-3’; and mutation of the first E-box, 5’-AGAA**CA**CA**TG**GA-3’ → 5’-AGAA**TG**CA**GT**GA-3’. The full-length Tal1, full-length Tcf3 (for Fig. 8H), Flag-Tcf3 fusion, full-length LSD1 (WT), and C-terminal-deleted LSD1 (LSD1-ΔC) sequences were cloned and inserted into the GV712 vector. Twelve Flag-Tcf3 fusion plasmids were constructed in the GV712 vector: Flag-full length (1-651aa), Flag-truncation 1 (1-99aa), Flag-truncation 2 (99-651aa), Flag-truncation 3 (100-239aa), Flag-truncation 4 (210-651aa), Flag-truncation 5 (211-298aa), Flag-truncation 6 (296-651aa), Flag-truncation 7 (310-430aa), Flag-truncation 8 (430-651aa), Flag-truncation 9 (430-547aa), Flag-truncation 10 (547-651aa), and Flag control vector. OVA plasmids were purchased from Addgene and used for constructing cells with stable OVA expression, which were identified by flow cytometry.

Functional-grade anti-mouse (m) CD3ε (clone 145-2C11) and anti-mCD28 (clone 37.51) antibodies were obtained from BD Bioscience. Fluorescently labeled antibodies specific for mCD4, mCD8, mCD44, mCD62L, mCD45.1, mCD45.2, mCD45, mIL-2, mTNF-α, mIFN-γ, mouse granzyme B, mKi-67, mPD-1, mTim-3, mTcf1, and mCXCR5 were purchased from Biolegend. The antibody against PD-1 (clone RMP1-14) and control IgG used in the in vivo studies were obtained from BioXCell. The antibodies against Id2 (clone 4E12G5), Tcf3, Tal1, LSD1 (clone EPR6825), HDAC1 (clone EPR23847-170), HDAC2 (clone EPR5001) and histone H3 (dime H3K4, clone Y47) were purchased from Abcam. The anti-GFP antibody (clone B-2) was purchased from Santa Cruz. The anti-Flag antibody (clone D6W5B) was purchased from CST. The antibodies against Actin (clone B4-B2), GAPDH and total histone H3 (6-A7) were purchased from HuaBio. Goat anti-hamster IgG (H + L) was purchased from Thermo Fisher.

Recombinant mouse IL-2 was obtained from PeproTech. PMA and ionomycin were obtained from Biolegend. The fluorescent dye CFSE was purchased from Sigma, and the nuclear dye Hoechst was purchased from Beyotime.

### Lentiviral infection and plasmid transfection

Lentiviral particles were generated using the indicated expression vectors, which were based on GV218-GFP or GV348-3×Flag-mCherry. Jurkat or HEK-293 cells were infected with lentiviruses and then cultured under the puromycin selection pressure. Transfection of the indicated plasmids was performed using Lipofectamine 2000 reagent (Invitrogen) according to the manufacturer’s instructions. Empty vectors were used as negative controls.

### Confocal immunofluorescence microscopy

After anti-CD3 antibody-induced activation, GFP-Id2^WT^ and GFP-Id2^ΔHLH^ Jurkat cells were stained with Hoechst and then added dropwise onto coverslips. The cells were observed with a confocal laser scanning microscope (Zeiss, Germany). Similarly, after PMA plus ionomycin stimulation, GFP-Id2^WT^ or GFP-Id2^ΔHLH^ HEK-293 cells were stained with Hoechst and observed with a confocal laser scanning microscope (Nikon, Japan).

### Dual luciferase reporter assay

For the dual luciferase reporter assay, HEK-293 cells were grown to 60–80% confluence. Slamf6 promoter-reporter gene constructs were then cotransfected with the following vectors using Lipofectamine 2000 (Invitrogen) according to the manufacturer’s instructions: for the experiment shown in Fig. 7A, Id2 (WT)-GV219, Id2 (ΔHLH)-GV219 and Tcf3-GV219; for the experiment shown in Fig. 8H, Tcf3-GV712, Tal1-GV712, LSD1 (WT)-GV712 and LSD1 (ΔC)-GV712. The amount of total DNA in each transfection reaction was normalized by the addition of the indicated empty vectors to the reaction mixture. The transfection efficiency was controlled by cotransfection of a plasmid constitutively expressing Renilla luciferase. For stimulation, cells were incubated with PMA plus ionomycin for the last 4 h after transfection. The cells were then used for the luciferase assay using the Dual-Luciferase Reporter Assay System (Promega). Relative luciferase activity was defined as the ratio of firefly luciferase activity to Renilla luciferase activity.

### Coimmunoprecipitation (Co-IP) and immunoblotting

GFP-Id2^WT^ and GFP-Id2^ΔHLH^ Jurkat cells were generated by lentiviral infection. HEK-293 cells were transfected with the appropriate plasmids. For Co-IP, a Pierce coimmunoprecipitation kit was used. Briefly, cells were harvested and lysed with IP lysis buffer containing 0.025 M Tris, 0.15 M NaCl, 0.001 M EDTA, 1% NP-40 and 5% glycerol (Thermo Fisher). Cell lysates were then incubated with the indicated antibody (anti-GFP, Santa Cruz; anti-Flag, CST Signaling; anti-Tcf3, Abcam)-conjugated resin in spin columns (Thermo Fisher) overnight at 4 °C with rotation. The resin was then washed with IP lysis buffer two times, and proteins were eluted with elution buffer. The eluted samples were then used for immunoblotting with appropriate antibodies. The immunoblot images were obtained in a Bio-Rad ChemiDoc Imaging System.

### Pull-down assay and LC/MS analysis

Jurkat cells were harvested and lysed with IP lysis buffer containing 0.025 M Tris, 0.15 M NaCl, 0.001 M EDTA, 1% NP-40 and 5% glycerol (Thermo Fisher). Cell lysates were then incubated with anti-Tcf3 antibody-conjugated resin in spin columns (Thermo Fisher) overnight at 4 °C with rotation. The resin was then washed with IP lysis buffer two times, and proteins were eluted with elution buffer. The eluted protein samples were then separated by SDS‒PAGE, and excised gel segments were subjected to mass spectrometry analysis to identify the Tcf3 interactome.

### Chromatin fractionation

For chromatin fractionation, a nuclear and cytoplasmic extraction kit (Thermo Fisher) was used to isolate the nuclear and cytoplasmic fractions from GFP-Id2^WT^ and GFP-Id2^ΔHLH^ Jurkat cells. The first two reagents listed in the kit instructions were added to the cell pellet for disruption of the cell membrane and release of cytoplasmic contents. After recovering the intact nuclei from the cytoplasmic extract by centrifugation, the nuclear proteins were extracted from the nuclei with the third reagent.

### ATAC-seq

CD8^+^ T cells were isolated from the spleens of *Id2*^fl/fl^*Cd4*-Cre^+^ mice and their littermates. Nuclear suspensions of these cells were incubated in transposition mix at 37 °C for 30 min. The products were immediately purified using a QIAGEN MinElute kit and sequenced using the Illumina HiSeq 4000 platform (or other platforms) by Gene Denovo Biotechnology Co. (Guangzhou, China).

### Chromatin immunoprecipitation (ChIP) and ChIP-seq

For ChIP, an EZ-Magna ChIP A/G chromatin immunoprecipitation kit (Millipore) was used to map the in vivo distribution of transcription factors associated with chromosomal DNA. Briefly, GFP-Id2^WT^ and GFP-Id2^ΔHLH^ Jurkat cells were first treated with formaldehyde to crosslink proteins to DNA to ensure coprecipitation. Cells were then lysed and sonicated to shear the chromatin into fragments of 200–1000 bp, and the fragment length was confirmed by gel electrophoresis. Subsequently, immunoprecipitation was conducted by incubation with a primary antibody (anti-Flag, anti-Tal1 or anti-H3K4me2) in combination with protein A/G-conjugated magnetic beads overnight at 4 °C with rotation. Protein‒DNA crosslinks were reversed, and DNA was purified to remove chromatin proteins. The purified DNA fragments were analyzed by gel electrophoresis and real-time quantitative PCR. H3K4me2 scores from ChIP-seq data of Jurkat and K562 cells were obtained from Cistrome DB [56, 57]. For ChIP-seq, transferred CD8^+^ T cells in B16-OVA tumors of recipient mice were isolated by flow cytometry. Library products with a size of 200–500 bp were enriched, quantified and sequenced on the NovaSeq 6000 platform (Illumina) in PE150 mode. ChIP-seq and data analysis were performed by Seqhealth Technology Co., Ltd. (Wuhan, China).

### Statistical analysis

GraphPad Prism 8 software was used for statistical analyses. Differences between two groups were assessed using Student’s *t* test. *P* values of less than 0.05 were considered to indicate a significant difference, and the level of significance is indicated as *P < 0.05, **P < 0.01, ***P < 0.001, or ****P < 0.0001. To compare mouse survival, the log-rank (Mantel‒Cox) test was performed.

### Study approval

All animal experiments were performed in accordance with protocols approved by the Ethics Committee of the National Translational Science Center for Molecular Medicine.

### Supplementary information

Supplementary material

Unedited blot and gel images
