# Supplementary material for: Id2 epigenetically controls CD8+ T-cell exhaustion by disrupting the assembly of the Tcf3-LSD1 complex
Source: Cell Mol Immunol. 2024 Jan 29;21(3):292–308. doi: 10.1038/s41423-023-01118-6 (PMC10902300; doi:10.1038/s41423-023-01118-6)
Supplement: Supplementary file 1 — Supplementary material [file 41423_2023_1118_MOESM1_ESM.docx]

**Supplementary material**

**Id2 epigenetically controls CD8^+^ T-cell exhaustion by disrupting the assembly of the Tcf3-LSD1 complex**

Yiming Li^1,2#^, Mingwei Han^1,2#^, Haolin Wei^1,2^, Wan Huang^1,2^, Zhinan Chen^1,2^, Tianjiao Zhang^1,2^, Meirui Qian^1,2^, Lin Jing^1,2^, Gang Nan^1,2^, Xiuxuan Sun^1,2^, Shuhui Dai^1,2^, Kun Wang^1,2^, Jianli Jiang^1,2*^, Ping Zhu^1,2*^, Liang Chen^2,3*^

^1^Department of Cell Biology of National Translational Science Center for Molecular Medicine and Department of Clinical Immunology of Xijing Hospital, Fourth Military Medical University, Xi’an, Shaanxi, 710032, China

^2^State Key Laboratory of New Targets Discovery and Drug Development for Major Diseases, Ganzhou, Jiangxi, 341000; Xi’an, Shaanxi, 710032, China

^3^School of Medicine, Shanghai University, Shanghai, 200444, China

^#^These authors contributed equally to this study.

**Corresponding Authors:**

Liang Chen, MD, PhD

E-mail: lchen1@shu.edu.cn

Ping Zhu, MD, PhD

E-mail: zhuping@fmmu.edu.cn

Jianli Jiang, MD, PhD

E-mail: jiangjl@fmmu.edu.cn


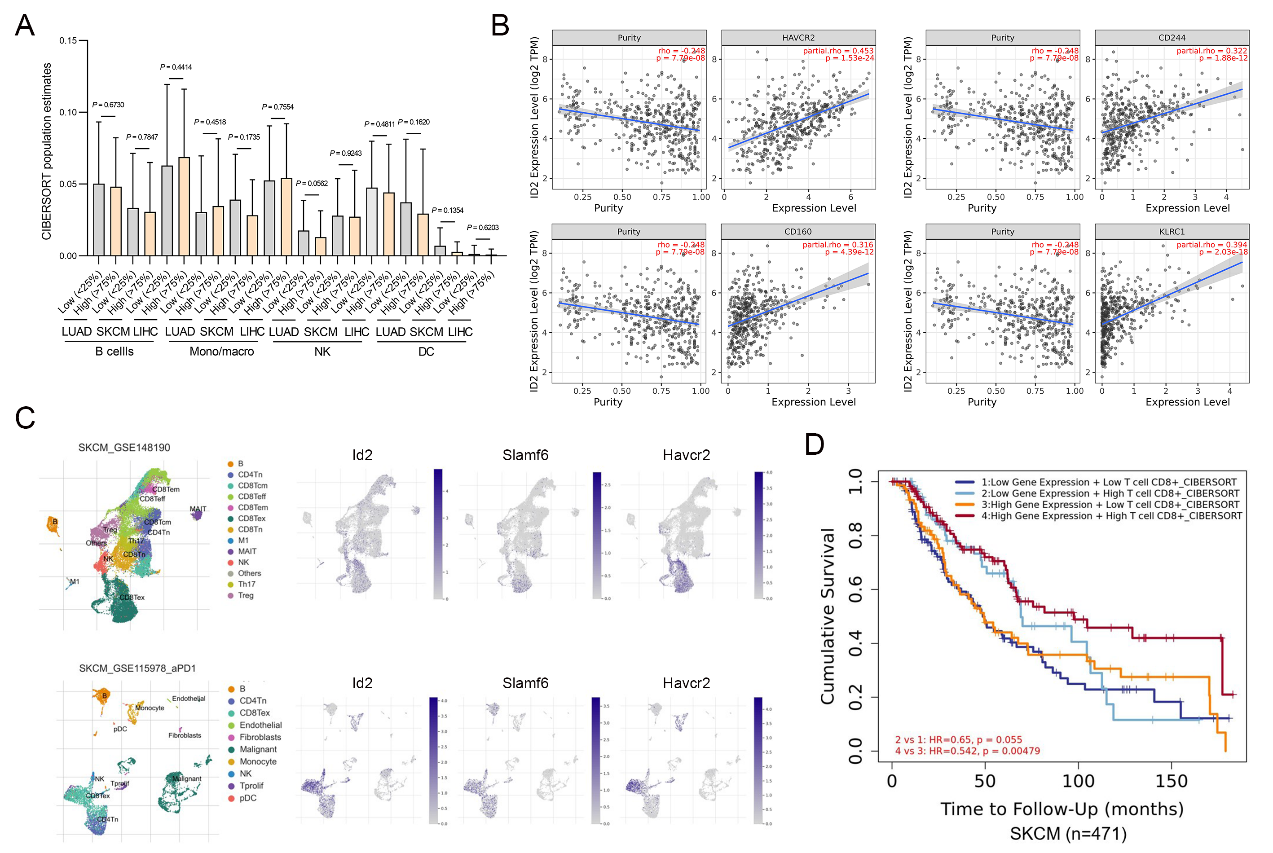


**Supplementary Figure 1. Id2 is selectively upregulated in tumor-infiltrating CD8^+^ T cells.** (**A**) Relative abundances of tumor-infiltrating immune cell populations determined by the CIBERSORT tool in LUAD, SKCM and LIHC patients with varying Id2 expression levels based on RNA-seq data in TCGA. LUAD, lung adenocarcinoma, *n*=532; SKCM, skin cutaneous melanoma, *n*=102; LIHC, liver hepatocellular carcinoma, *n*=370. (**B**) Correlations of *Id2* expression with the expression of inhibitory receptors (*Cd244, Havcr2, Cd160* and *Klrc1*) in SKCM. (**C**) Id2 expression profiles visualized on a tSNE plot of major immune cell types in the SKCM TME. The GSE148190 dataset contains data from 3 patients (27834 cells). GSE115978 dataset (treated with anti-PD-1) contains data from 31 patients (7186 cells). (**D**) Kaplan-Meier plots of the overall survival among patients stratified by both the estimated infiltration level of CD8^+^ T cells and Id2 expression level in SKCM (n=471).


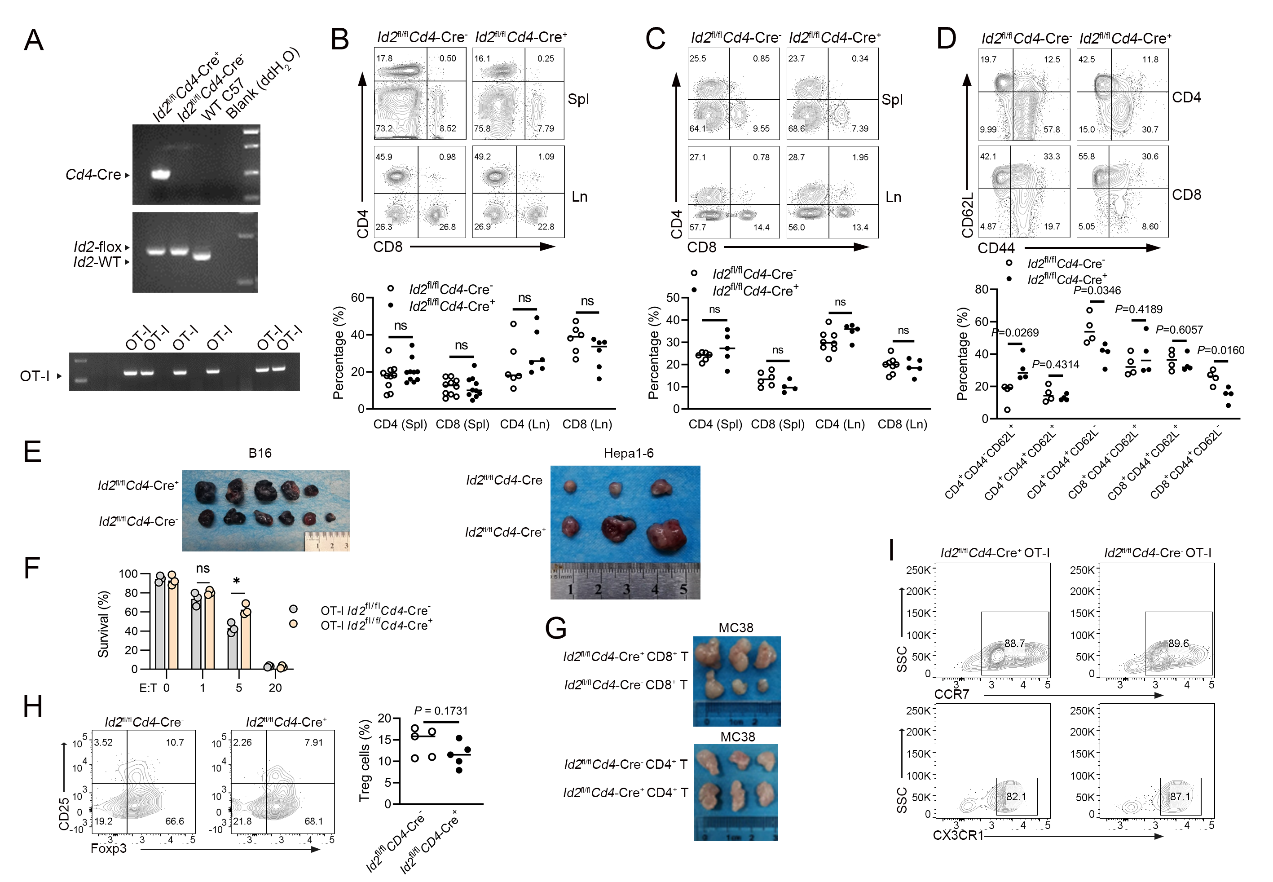


**Supplementary Figure 2. Deletion of Id2 in T cells promotes tumor development both in vivo and in vitro**. (**A**) Genotyping PCR of the alleles. Up panel, amplifying the *Cd4*-Cre, *Id2*-flox and *Id2*-WT cDNA; bottom panel, amplifying the OT-I cDNA. (**B**) Flow cytometric analysis of the frequencies of CD4^+^ T cells and CD8^+^ T cells in both spleens and lymph nodes of *Id2*^fl/fl^*Cd4*-Cre^-^ mice and *Id2*^fl/fl^*Cd4*-Cre^+^ mice without PMA stimulation. Spl, spleen; Ln, lymph node. (**C**) Flow cytometric analysis of the frequencies of CD4^+^ T cells and CD8^+^ T cells in both spleens and lymph nodes of *Id2*^fl/fl^*Cd4*-Cre^-^ mice and *Id2*^fl/fl^*Cd4*-Cre^+^ mice with PMA stimulation. (**D**) Flow cytometric analysis of the frequencies of CD44^+^CD62L^+^, CD44^+^CD62L^-^ and CD44^-^CD62L^+^ CD4^+^ T cells or CD8^+^ T cells in spleens of *Id2*^fl/fl^*Cd4*-Cre^-^ mice and *Id2*^fl/fl^*Cd4*-Cre^+^ mice. (**E**) Gross evaluations of tumor development. Left panel, B16; right panel, Hepa1-6. (**F**) Quantification of Hepa1-6^GFP^-SIINFEKL cells following coculture with CD8^+^ T cells derived from *Id2*^fl/fl^*Cd4*-Cre^-^ OT-I mice or *Id2*^fl/fl^*Cd4*-Cre^+^ OT-I mice. The data are presented as summary graphs. ns, not significant; *, *P <* 0.05. (**G**) Gross assessments of tumor development in the MC38-bearing Rag1^-/-^ model. (**H**) Flow cytometric analysis of the frequencies of Tregs in MC38 tumors of *Id2*^fl/fl^*Cd4*-Cre^-^ mice and *Id2*^fl/fl^*Cd4*-Cre^+^ mice. (**I**) Assessment of CCR7 and CX3CR1 in adoptively transferred *Id2*^fl/fl^*Cd4*-Cre^-^ or *Id2*^fl/fl^*Cd4*-Cre^+^ OT-I CD8^+^ T cells in B16-OVA tumors.


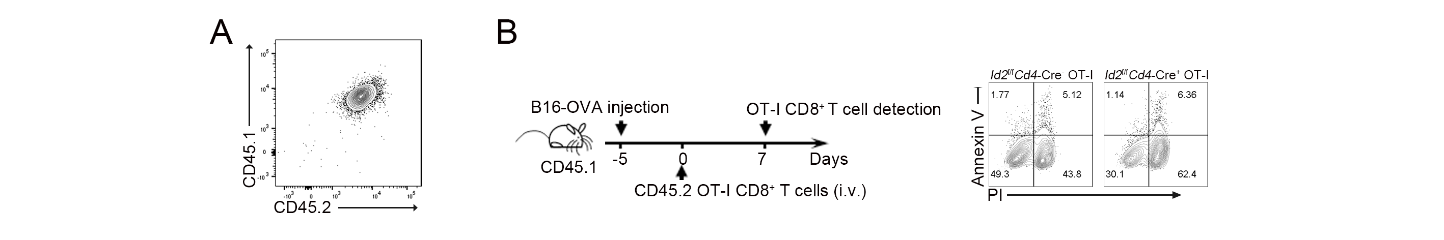


**Supplementary Figure 3.** Identification of CD45.1.2 OT-I Id2^WT^ mice and assessment of the effect of Id2 on CD8^+^ T-cell apoptosis. (**A**) Flow cytometry identification of CD45.1.2 OT-I Id2^WT^ mice. (**B**) Assessment of apoptosis of adoptively transferred OT-I CD8^+^ T cells in B16-OVA tumors.

**
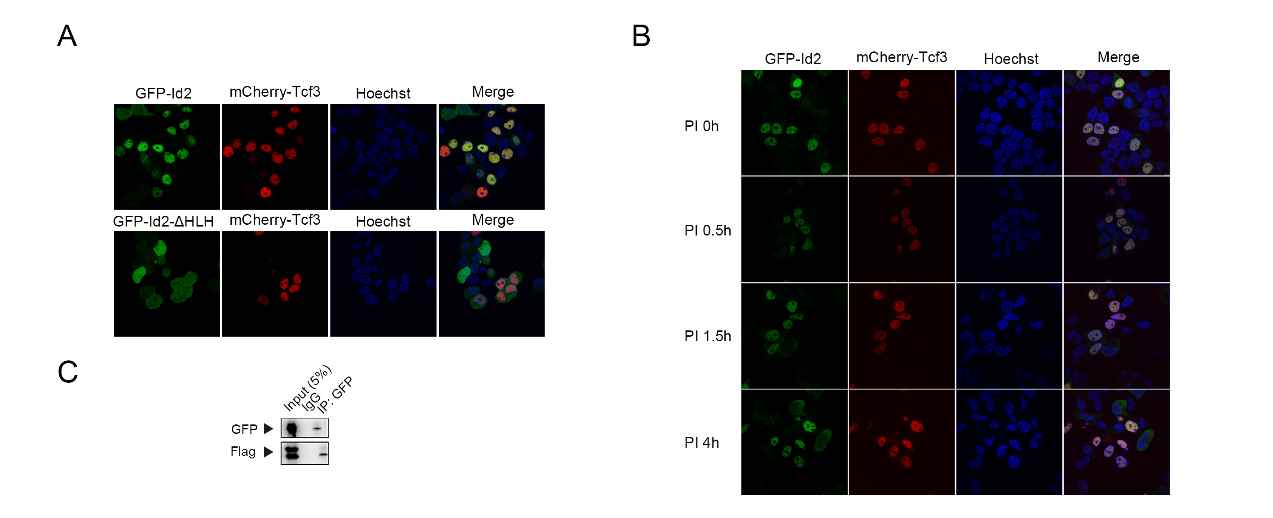
**

**Supplementary Figure 4. Confocal or co-immunoprecipitation examination of Id2-Tcf3 interaction.** (**A**) Confocal examination of GFP-Id2 and mCherry-Tcf3 localization in GFP-Id2^WT^ or GFP-Id2^ΔHLH^ HEK-293 cells. (**B**) Confocal examination of GFP-Id2 and mCherry-Tcf3 localization in HEK-293 cells with PMA plus ionomycin stimulation. (**C**) Co-immunoprecipitation (IP) analysis of Id2-Tcf3 interaction in HEK-293 cells. GFP, GFP-Id2; Flag, Flag-Tcf3.

**
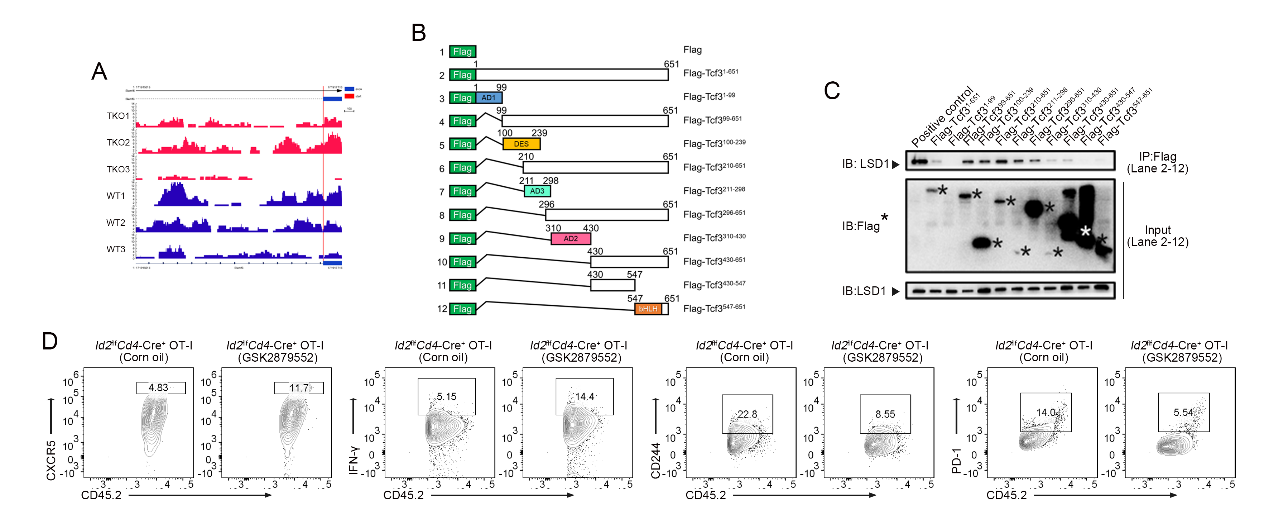
**

**Supplementary Figure 5.** (**A**) ATAC-seq of *Slamf6* promoter (TSS upstream 2000 to downstream 200). (**B**) Schematic representation of full-length Tcf3 and its deletion mutants lacking functional domains. (**C**) Co-immunoprecipitation (IP) analysis of the interaction of LSD1 and full-length Tcf3 or its deletion mutants in HEK-293 cells. (**D**) Assessment of CXCR5, IFN-γ, CD244 and PD-1 expression of *Id2*^fl/fl^*Cd4*-Cre^+^ CD8^+^ T cells with corn oil or GSK2879552 in MC38-OVA tumors.


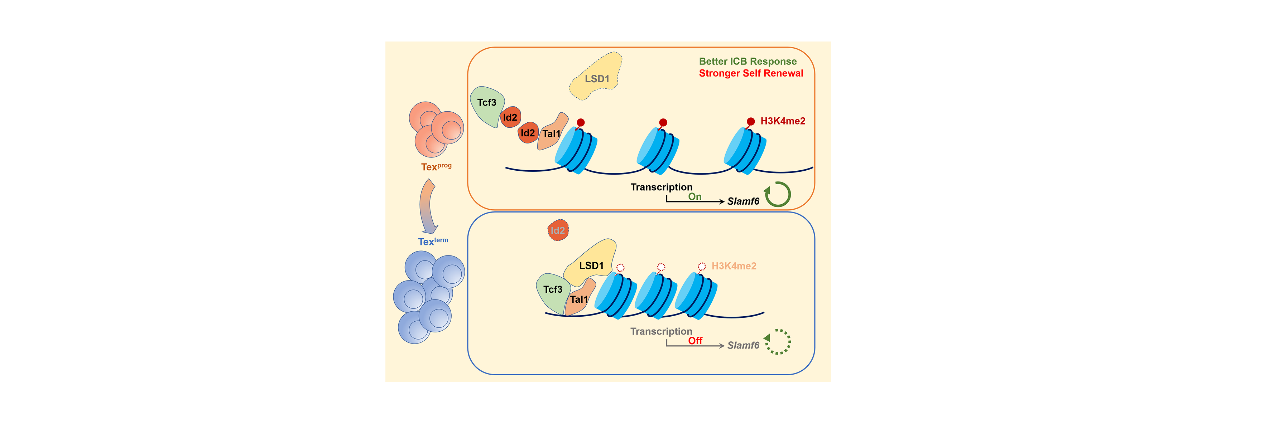


**Supplementary Figure 6.** Model for the role of Id2-mediated transcriptional and epigenetic modification in T-cell exhaustion.
