# Supplementary figures and images for: Id2 epigenetically controls CD8+ T-cell exhaustion by disrupting the assembly of the Tcf3-LSD1 complex
Source: Cell Mol Immunol. 2024 Jan 29;21(3):292–308. doi: 10.1038/s41423-023-01118-6 (PMC10902300; doi:10.1038/s41423-023-01118-6)

7C

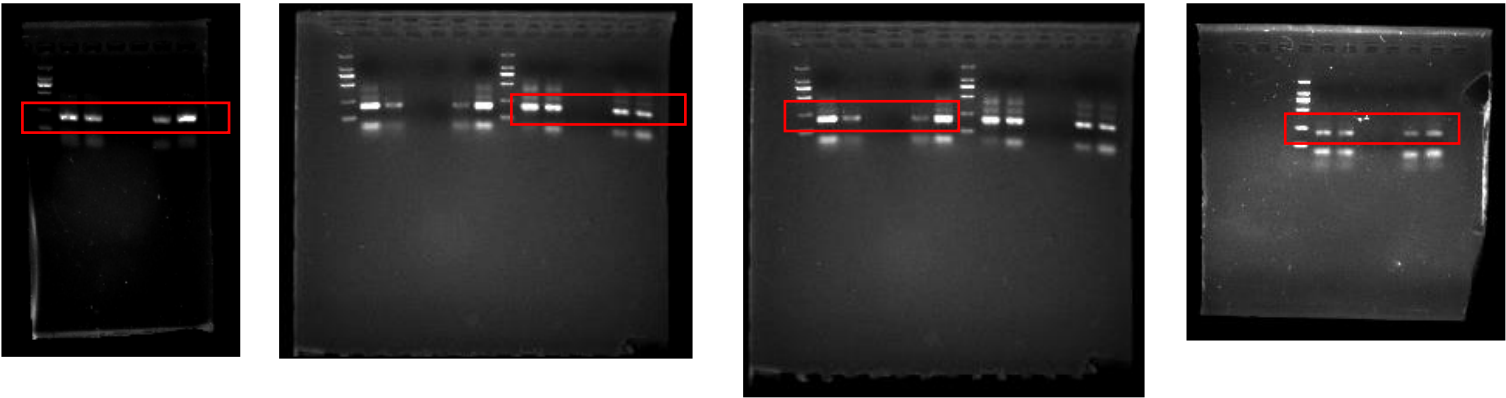

7E

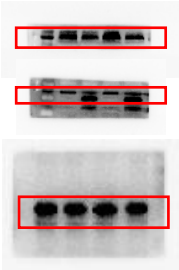

7I

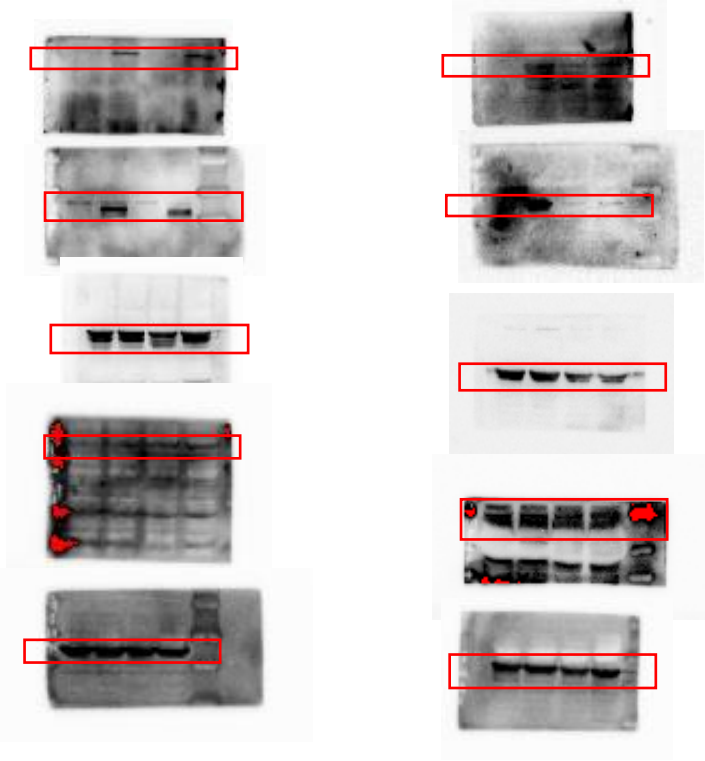

7H

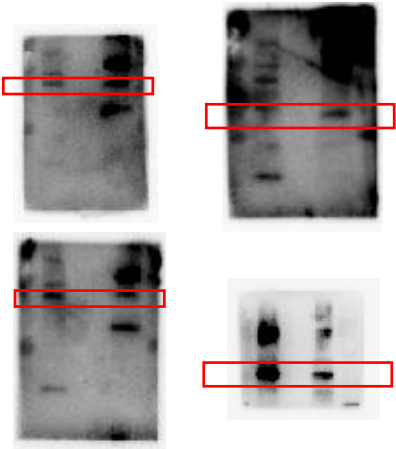

7J

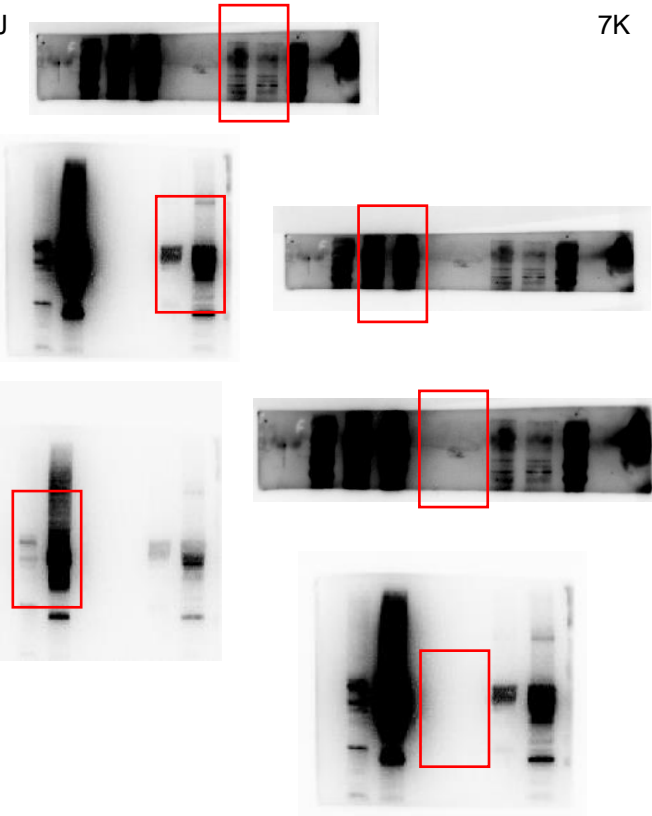

7K

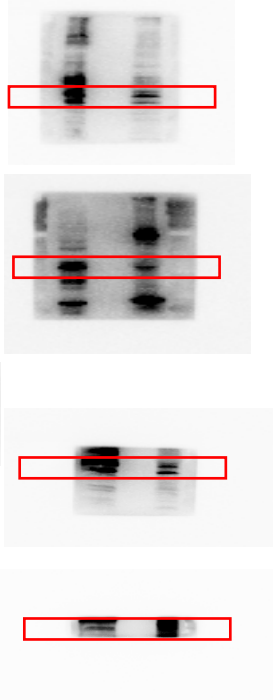

7L

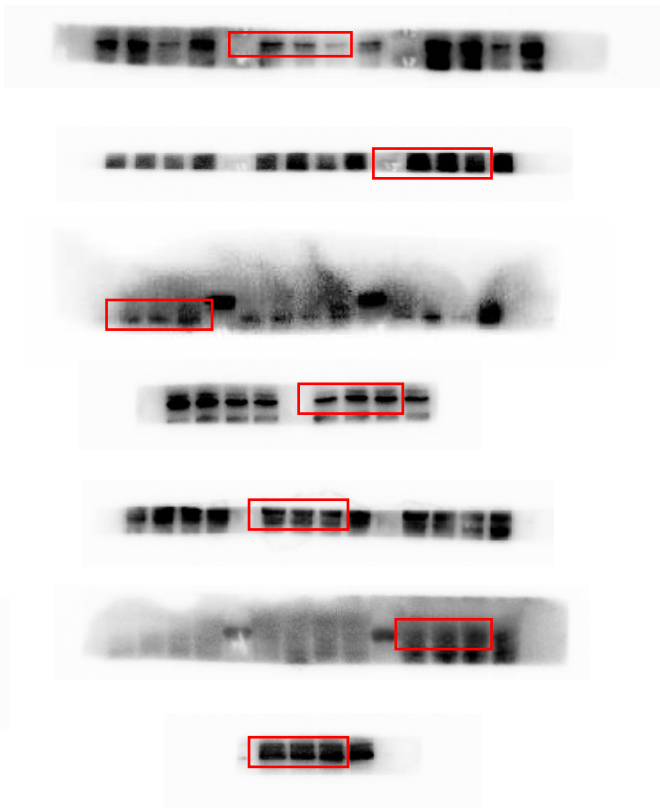

8D

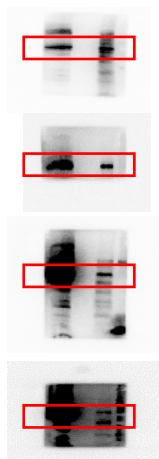

8E

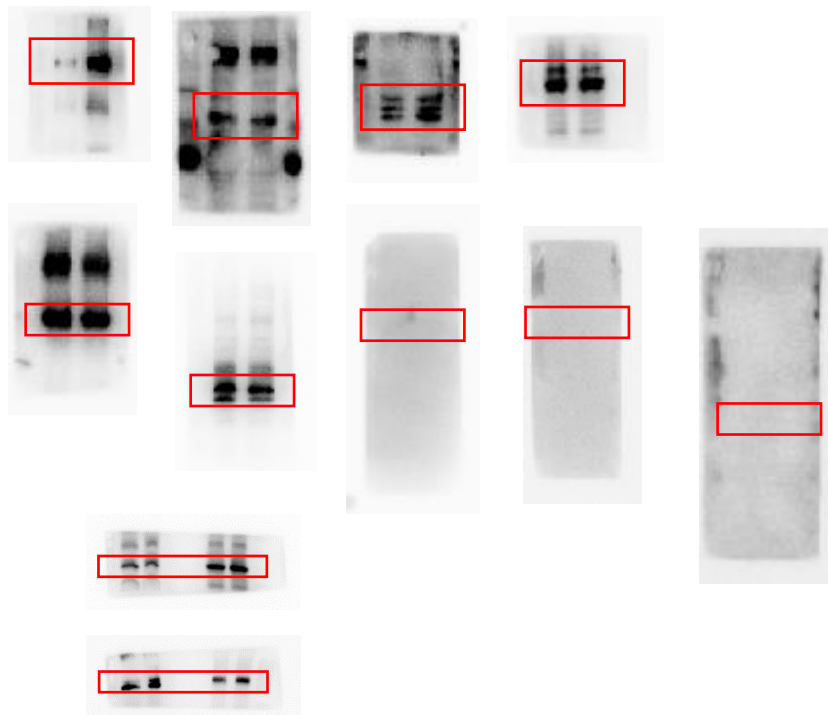

8F

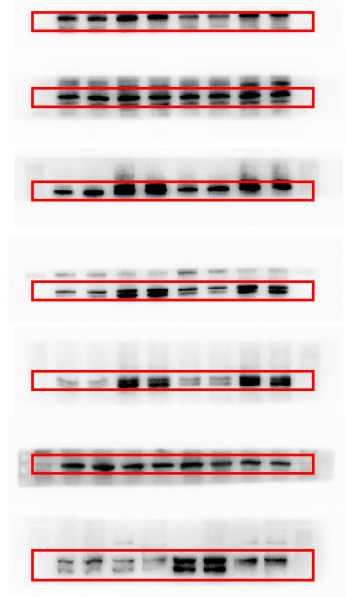

8G

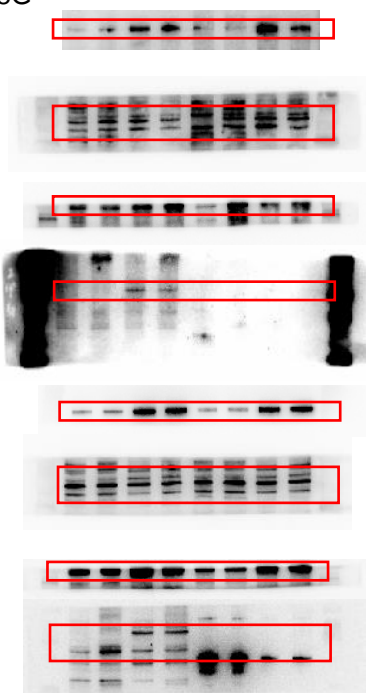

8I

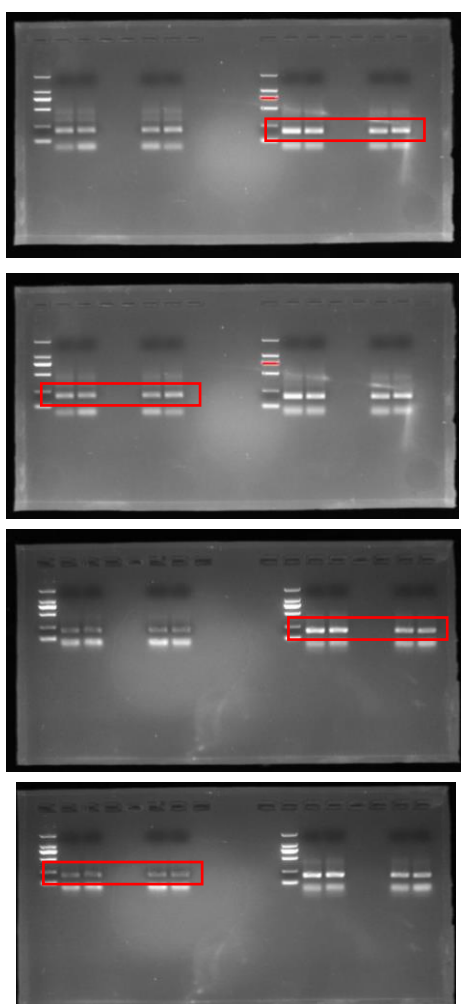

8L

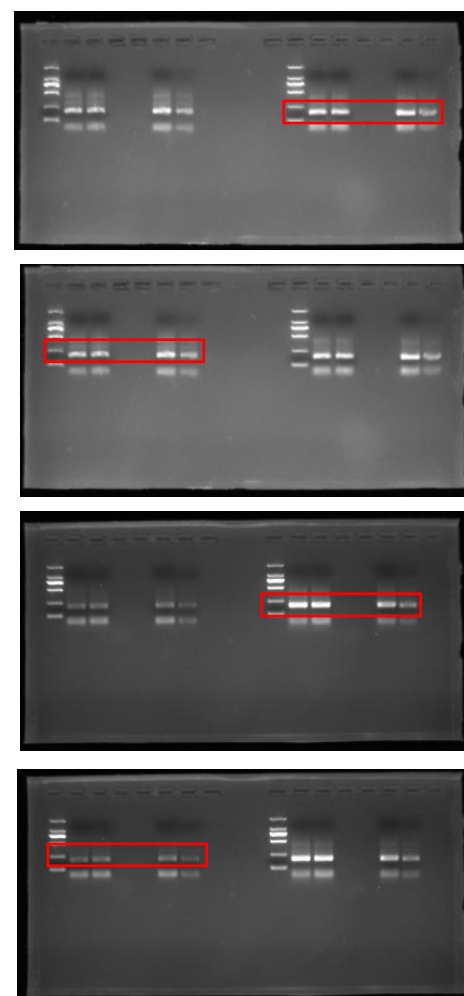

8K

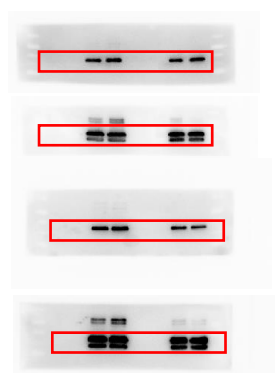

S2A

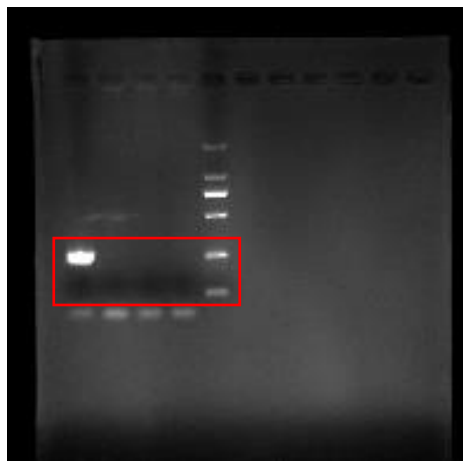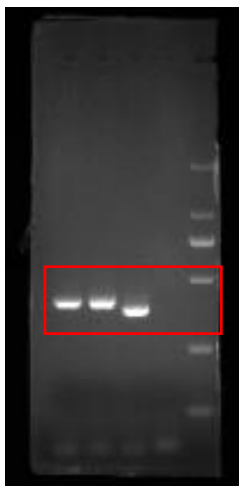

S4C

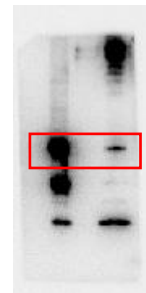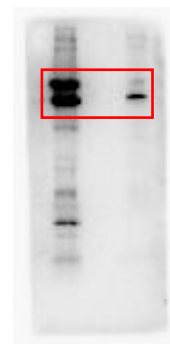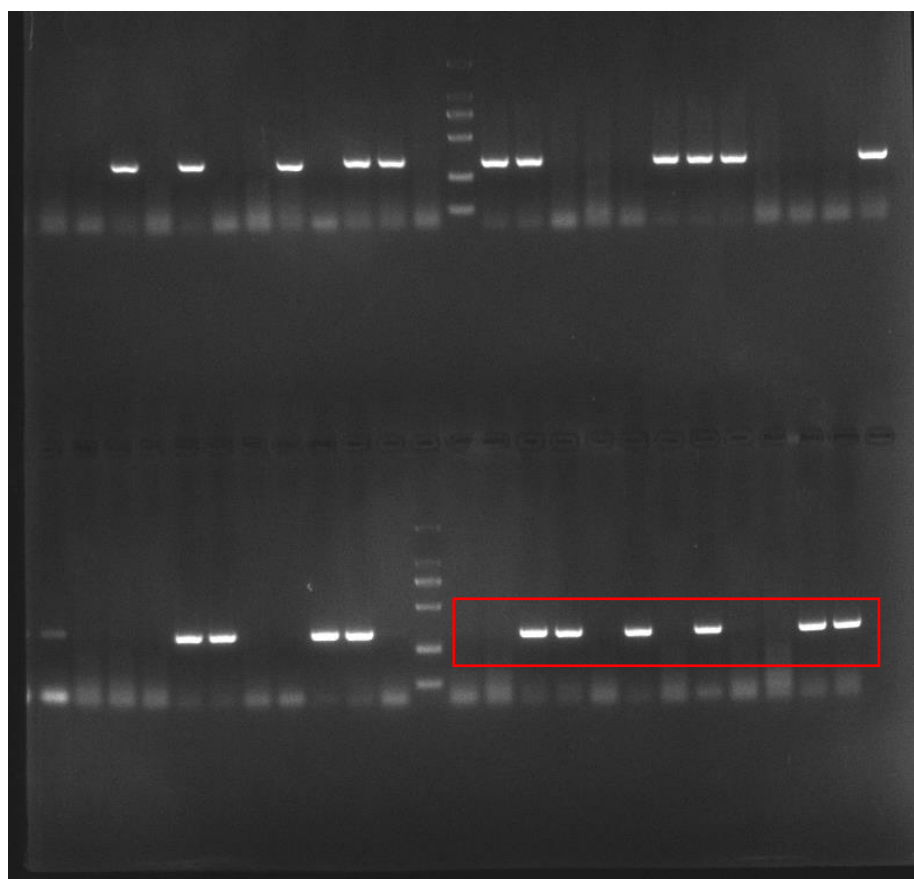

S5C

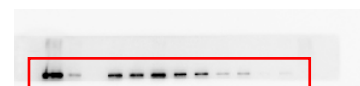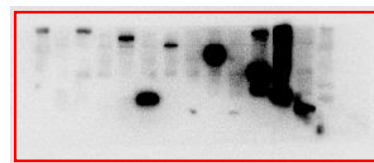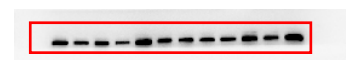

Supplement: Supplementary file 2 — Unedited blot and gel images [file 41423_2023_1118_MOESM2_ESM.pdf]
